# Non-pharmacological interventions for the improvement of post-stroke activities of daily living and disability amongst older stroke survivors: A systematic review

**DOI:** 10.1371/journal.pone.0204774

**Published:** 2018-10-04

**Authors:** Carrie Stewart, Selvarani Subbarayan, Pamela Paton, Elliot Gemmell, Iosief Abraha, Phyo Kyaw Myint, Denis O’Mahony, Alfonso J. Cruz-Jentoft, Antonio Cherubini, Roy L. Soiza

**Affiliations:** 1 Department of Old Age Medicine, Aberdeen Royal Infirmary, Aberdeen, United Kingdom; 2 School of Medicine & Dentistry, University of Aberdeen, Aberdeen, United Kingdom; 3 Geriatria, Accettazione geriatrica e Centro di ricerca per l’invecchiamento, IRCCS INRCA, Ancona, Italy; 4 Department of Geriatric Medicine, University College Cork, Cork, Ireland; 5 Fundación para la Investigación Biomédica del Hospital Universitario Ramón y Cajal, Madrid, Spain; Cardiff University, UNITED KINGDOM

## Abstract

Globally, stroke remains a leading cause of death and disability, with older adults disproportionately affected. Numerous non-pharmacological stroke rehabilitation approaches are in use to address impairments, but their efficacy in older persons is largely unknown. This systematic review examined the evidence for such interventions as part of the Optimal Evidence-Based Non-Drug Therapies in Older Persons (ONTOP) project conducted under an European Union funded project called the Software Engine for the Assessment and Optimisation of Drug and Non-Drug Therapies in Older Persons (SENATOR) [http://www.senator-project.eu]. A Delphi panel of European geriatric experts agreed activities of daily living and disability to be of critical importance as stroke rehabilitation outcomes. A comprehensive search strategy was developed and five databases (Pubmed, CINAHL, Embase, PsycInfo and Cochrane Database of Systematic Reviews) searched for eligible systematic reviews. Primary studies meeting our criteria (non-pharmacologic interventions, involving stroke survivors aged ≥65 years, assessing activities of daily living and/or disability as outcome) were then identified from these reviews. Eligible papers were double reviewed, and due to heterogeneity, narrative analysis performed. Cochrane risk of bias and GRADE assessment tools were used to assess bias and quality of evidence, allowing us to make recommendations regarding specific non-pharmacologic rehabilitation in older stroke survivors. In total, 72 primary articles were reviewed spanning 14 types of non-pharmacological intervention. Non-pharmacological interventions based on physiotherapy and occupational therapy techniques improved activities of daily living amongst older stroke survivors. However, no evidence was found to support use of any non-pharmacological approach to benefit older stroke survivors’ disability. Evidence was limited by poor study quality and the small number of studies targeting older stroke survivors. We recommend future studies explore such interventions exclusively in older adult populations and improve methodological and outcome reporting.

## Introduction

Globally, stroke remains a leading cause of death and disability, with older adults disproportionately affected[1–2]. While effective acute treatment has increased stroke survival within developed nations, increased survival increases the number of those affected by post-stroke impairments [2]. Therefore, effective rehabilitation which can reduce post-stroke impairment and restore a person’s functional abilities is imperative.

Stroke guidelines recommend utilising multi-disciplinary stroke rehabilitation teams [3–5]. This reflects the diverse physical, psychological and social rehabilitation needs of stroke survivors [3–5]. Rehabilitation is primarily non-pharmacologic in nature, and standard approaches include occupational therapy (OT), physiotherapy (PT), and speech therapy [3–5]. Several factors contribute towards the overall success of stroke rehabilitation and include stroke severity, the type and location of a stroke, and the patient’s general health and pre-stroke health [6]. The patient’s age is also generally accepted to be highly influential; older patients are at a higher risk of poorer outcomes following stroke rehabilitation [7].

The evidence base for many of non-pharmacologic stroke rehabilitation interventions is poor. For example, the National Clinical Guidelines for stroke [4], despite recommending that psychological care be offered to all stroke survivors, also describe the evidence behind many psychological therapies (e.g. Cognitive Behavioural Therapy, Counselling) as conflicting and inconclusive. Furthermore, the effectiveness of such interventions within the older stroke population is even less clear. Much of the literature reports upon trials involving participants below 65 years of age [8]. Older adults may differ from younger adults in terms of their rehabilitation needs and preferences [8]. Ageing brings more challenges; older stroke survivors often have higher pre- and post-stroke disability and impairments, some of which can be explained though the natural ageing process [9]. This may make rehabilitation more challenging, limiting benefits from rehabilitation attempts.

While non-pharmacological approaches to treat post-stroke impairments are predominant, they are also preferred for older patients. Older people have an increased risk of adverse drug reactions [10]. Additionally, many drugs commonly prescribed to older people have not been assessed in an older population [8]. Therefore, pharmacologic agents used to treat some post-stroke impairment, such as muscle spasticity and movement disorders, are unlikely to have been adequately tested in older patients and so their safety amongst this population group is unknown.

There are compelling reasons behind treating common conditions using non-drug therapies amongst older persons. The aim of this systematic review was to identify and review the evidence for such interventions as applied to older stroke survivors. This systematic review is part of the Optimal Evidence-Based Non-Drug Therapies in Older Persons (ONTOP) project. The ONTOP project aims to systematically review 15 of the most prevalent and difficult to manage conditions in older people and produce a list of recommendations concerning the use of non-drug therapies for these conditions [11–12]. Many of these reviews have been completed, including for pressure ulcer risk reduction and treatment [13] reduction in incidence and treatment of delirium [12] and fall prevention [14]. ONTOP is in turn part of a larger, European Union (EU) funded project called the Software Engine for the Assessment and Optimisation of Drug and Non-Drug Therapies in Older Persons (SENATOR) [11]. Recommendations from ONTOP reviews are intended for use in the SENATOR project to produce a software programme that can advise clinicians on the use of pharmacological and non-pharmacological therapies in older persons, while limiting the risk of polypharmacy and adverse drug reactions [12].

## Methods

**Fig 1:**
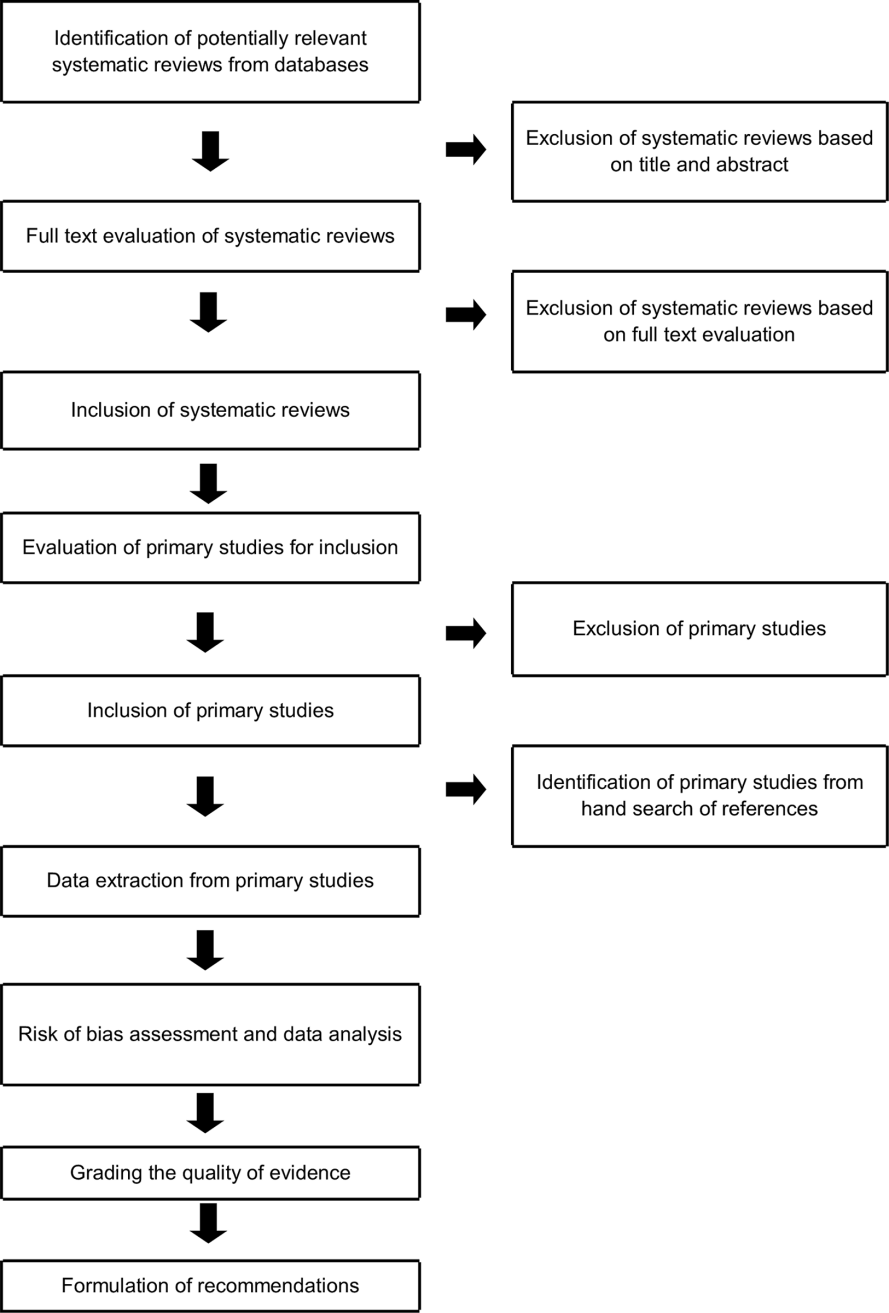
ONTOP review methodology.

The systematic review methodology was developed specifically for the ONTOP project. Fig 1 presents an outline of the stages this methodology involved. In summary, the methodology was devised to capture primary studies, RCTs or quasi-RCTs, from published systematic reviews. This process was followed in this review of non-pharmacological interventions for the treatment of older stroke survivors. Outcomes were determined by consensus opinion using the Delphi approach, as described below. Review protocol has not been registered but has been published [12].

### Delphi process

Outcomes were selected by a panel of 13 European experts in geriatric medicine using a Delphi process, a structured, questionnaire-based method of reaching consensus [15]. A literature review generated a list of all outcome measures used in stroke research which was then given to panellists as a questionnaire. Panellists, anonymously, rated each outcome from 1–9 according to their perception of its clinical importance. The mean score for each outcome was then used to categorise outcomes by importance: not important (score of 1–3), important but not critical (score of 4–6), and critically importance (7–9). These boundaries were selected based on the Grading of Recommendations, Development and Evaluation (GRADE) method for evaluating the quality of evidence [16]. Panel members could suggest additional outcomes for consideration if they felt that an important outcome had been overlooked. Outcomes ranked as critical were used for this review. Activites of daily living (ADL), quality of life and disability were the only outcomes rated as being critically important. For brevity, in this paper we present the results for ADL and disability only. The results for quality of life will be reported in a separate publication.

### Literature search strategy

A search strategy (Fig 1) was designed based on Montori’s highly specific search strategy for retrieving systematic reviews from PubMed [17]. This search strategy was then modified for use in other databases. In total, five databases were searched (Cinahl, Cochrane Database of systematic Reviews, Embase, PsycInfo, PubMed) without restrictions on publication status or date. The search strategy is presented as supplementary material (S1 Table).

### Inclusion criteria

#### Systematic reviews

- Full text was available in English, Spanish or Italian.
- Identified at least one primary study matching this review’s inclusion criteria.
- Specifically mentioned conducting a search of at least one medical literature database.
- Guidelines were also considered for inclusion provided that they used a transparent and systematic approach to retrieve the evidence.

#### Primary studies

- All participants must be ≥65 years of age, or the mean age of participants must be ≥65 years of age
- All aetiologies, types and severity of stroke/ stroke symptoms included
- Involves any non-pharmacological intervention for stroke:
  - a single or multi-component non-drug intervention used to improve symptoms post-stroke
  - a non-drug intervention being a treatment or therapy that can be performed on or given to a patient, and/or taught to the patient for them to practice themselves.
  - A non -drug intervention which is deliverable in clinical practice
- Treatment for any complications or specific disability of stroke (e.g. urinary incontinence, shoulder subluxation, neglect syndrome etc.) will be included **if** the study reports ≥1 relevant outcome
- Compares the non-pharmacologic treatment against no treatment, a sham intervention or a treatment considered standard practice at the time of the study.
- A study using Randomised Controlled Trial (RCT) or Quasi RCT methodology
- Paper must focus on at least one or more of three Delphi consensus derived outcome variables: ADL, quality of life or disability (global measures only).
- Papers published only in English, Italian and Spanish

### Exclusion criteria

#### Primary studies

- Any therapy for *stroke prevention*
- Any therapy using non-conventional products but administered in a conventional route (e.g. Chinese medicine, herbal supplements)
- Observational or before-after studies with historical controls
- The inclusion of participants with other neurological conditions
- Studies exploring the management of stroke in critical care/ Accident &Emergency
- Health services research evaluating the two different stroke units (hospital based, community or home-based), two or more different methods of delivering non-pharmacological therapy (e.g. face to face or telephone rehabilitation), or evaluating different methods of delivering/ co-ordinating discharge care (e.g. named person in charge of discharge/ post-discharge care versus usual care)
- Economic evaluations of non-pharmacological therapy
- Papers discussing the dose-response relationship (duration, intensity of therapy or time to commence treatment, including early discharge)
- Interventions which only involve the provision of education/ stroke information and general sign posting/ liaison with other services where the patient plays a passive role (NB: If these components are included in a broader structured multi-component intervention such as a self-management programme the intervention will be included).

### Study selection

**Fig 2:**
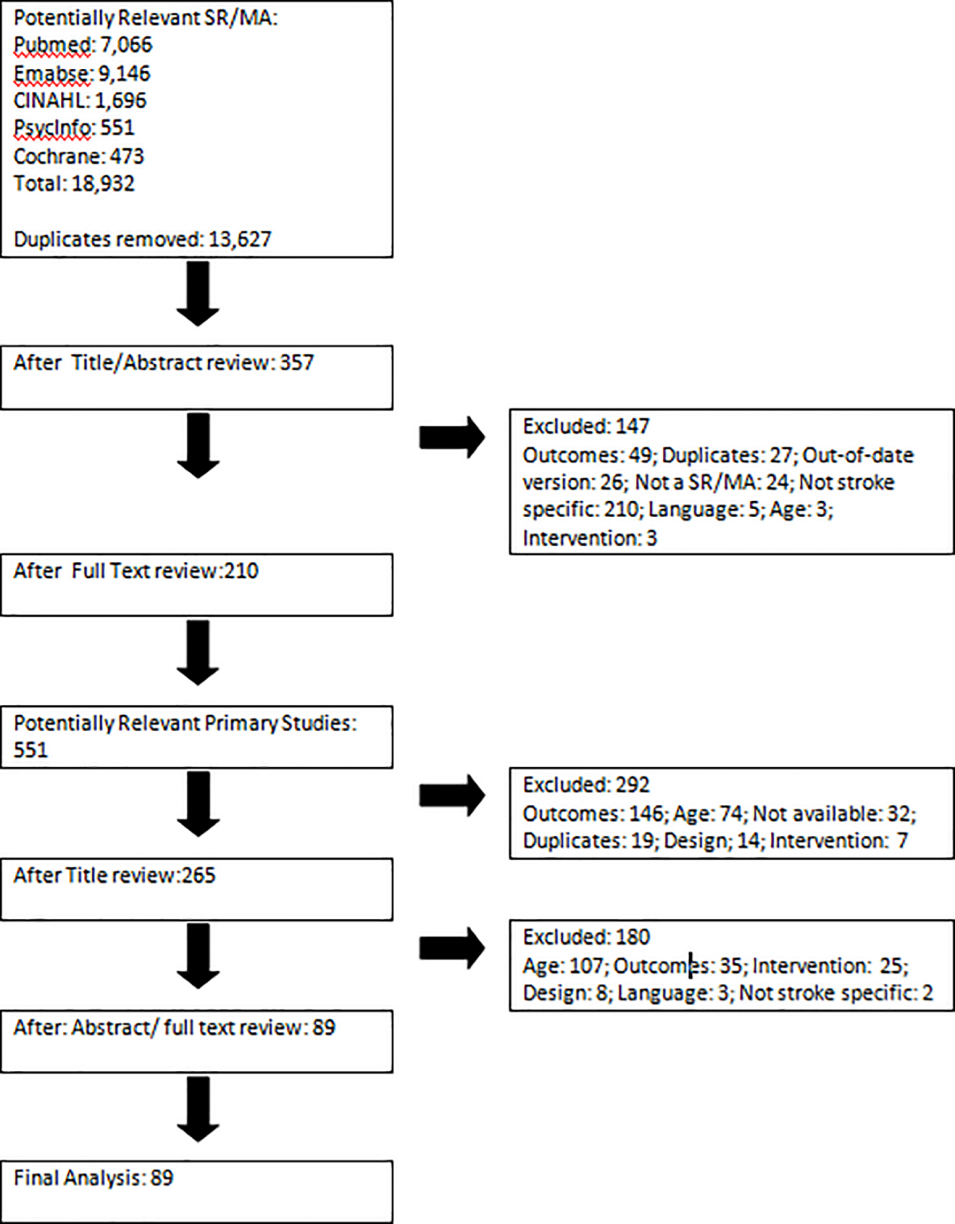
Study selection process.

For this review, 18,932 potentially relevant articles were identified from database searches (Fig 2). After removing duplicates, 13,627 unique records were screened by title and abstract by two reviewers. Only 363 full texts of systematic reviews were deemed eligible based on their abstracts. Of these, 173 reviews matched the eligibility criteria and were read in full, and their references were hand searched to identify potentially relevant primary studies. The initial searches were conducted in December 2015, with no restrictions on publication date, and resulted in 83 primary articles for inclusion. The review was updated as above in April 2018 and a further six papers were added to the findings.

### Data collection

The results of the database searches were amalgamated using Refworks 6.0 software (ProQuest LLC, USA). A list of the titles and abstracts of systematic reviews were screened by two independent assessors (EG, CS). Any disagreements over eligibility were resolved through discussion with other members of the research team (RS and PKM).

The full-text articles of potentially eligible reviews and meta-analyses were then retrieved and assessed for eligibility, again by two independent assessors (EG and CS). The references of the included studies in eligible systematic reviews were hand-searched to identify primary studies relevant to this review. A list of the titles and abstracts of potentially eligible primary studies was screened (EG, CS, SS, RS and PKM). Thereafter, the full-text articles of potentially relevant primary studies were retrieved and screened by EG and CS.

### Data extraction

A data extraction form was designed by adapting the Cochrane Collaboration’s Data Extraction and Assessment Template. The information contained on the data extraction forms (study methodology, participant characteristics, and outcome data) was then transferred to an Excel spreadsheet for narrative analysis. Results were also transferred to RevMan 5.3 [Cochrane Collaboration, UK, http://community.cochrane.org/help/tools-and-software/revman-5] to facilitate risk of bias tables. Results were also transferred to the GRADE Pro online system [http://www.gradeworkinggroup.org] for the development of recommendations for each type of non-pharmacological intervention. Types, or categories, for non-pharmacological interventions were developed and applied to organise the included studies into meaningful categories of interventions for the analysis. Data extraction was performed by two independent assessors (CS & EG).

### Risk of bias

Risk of bias was assessed using the Cochrane Collaboration’s Risk of Bias tool [18]. This tool assesses: random sequence generation, allocation concealment, blinding of participants and personnel, blinding of outcome assessment, incomplete outcome data, selective reporting, and other biases. A decision was made as to whether the risk of bias for each category should be described as low, unclear or high risk. The overall risk of bias for the study was then judged by taking account of the scores for each individual category. Results from the risk of bias assessment were entered into RevMan 5.3 software to enable the production of risk of bias graphs and summary tables.

### Development of PICO questions

Clinical questions were formulated using the PICO (Population, Intervention, Comparator, and Outcome) framework for each intervention type and outcome assessed. Due to the small number of papers in each category of intervention, the PICO questions chosen were considered to be the most pertinent and inclusive questions. For most categories of intervention one question assessing the efficacy of intervention types upon each outcome was chosen. As physiotherapy and occupational therapy are often standard care in stroke rehabilitation, studies investigating these therapies did not have a no intervention control. Therefore, we split physiotherapy and occupational therapy studies depending upon whether they compared a more intensive (increased time and duration) of therapy against usual intensity, or if they compared two or more different forms of therapy.

### Narrative analysis

All primary studies were included in a narrative assessment. The effects reported in each study were described as favouring the intervention, favouring the control, or as showing no significant difference. The overall findings of the studies were assessed qualitatively considering methodological quality and risk of bias. Patterns of effect across the studies were described and possible reasons for effect differences between studies explored, as per guidance offered by the ESRC [19]. Due to substantial clinical heterogeneity between studies and poor study reporting, meta-analysis of results was not considered appropriate. Clinical heterogeneity was assessed qualitatively by all authors and focused upon intervention content, target (e.g. upper or lower limb impairment), delivery, duration.

### Assessing quality of evidence

After the completion of analysis, evidence for each non-pharmacological category was assessed using the GRADE method [16]. The GRADE approach assesses the evidence across all studies analysed for a given outcome, rather than assessing the evidence from each study individually. The GRADE framework allows the quality of the body of evidence, and consequentially any recommendations to be made from this evidence, to be judged across five criterions known to limit the quality of evidence. Further details regarding each of these criteria can be found on the GRADE website [http://www.gradeworkinggroup.org]. The quality of the evidence was assigned an overall rating of quality, as described below in Table 1.

**Table 1:** GRADE evidence rating descriptions.

Quality Level	Description
**High quality**	*Further research is very unlikely to change our confidence in the estimate of effect*
**Moderate quality**	*Further research is likely to have an important impact on our confidence in the estimate of effect and may change the estimate*
**Low quality**	*Further research is very likely to have an important impact on our confidence in the estimate of effect and is likely to change the estimate*
**Very low quality**	*Any estimate of effect is very uncertain*

### Development of recommendations

After the quality of evidence had been determined, concise recommendations were designed regarding the use of non-pharmacological therapies after stroke in older persons. These recommendations were written taking account of the quantity, quality and GRADE score of the available evidence.

## Results

Of 89 retrieved articles, 72 papers included ADL and/ or Disability as an outcome measure. Results are presented below, organised by type of non-pharmacological intervention; Acupuncture (n = 11), Caregiver Training (n = 1), Constraint Induced Movement Therapy (CIMT, n = 2), Device-assisted Physiotherapy (n = 8), Music Therapy (n = 1), Nerve Stimulation (n = 3), Occupational Therapy (OT, n = 12), Optical Interventions (n = 3), Physiotherapy (n = 17), Psychological Therapies (n = 6), Self-management Education (n = 6), Videogames (n = 1), and Wheelchair (n = 1).

### Acupuncture

#### Studies

Eleven randomised controlled trials (RCT) were included in this category. Five were conducted in China, two in Sweden, two in the UK and one each from Taiwan and Germany.

#### Participants

In total, 1064 participants were involved. Mean ages ranged from 65.5 (SD 9.71) years [20] to 78.3 (SD 5.9) years [21]. Time between stroke onset and commencing intervention ranged from a mean of 14.2 (SD 19.2) hours [22] to 14.4 (SD 7.8) days [20]. Participant’s characteristics across the included studies are presented in Table 2.

**Table 2:** Participant characteristics and study descriptions of included acupuncture studies.

Study	Arm	No. of Participants	Male/ Female	Age (Yrs, Mean, SD)	Time post-stroke (Mean, SD)	Description	Timing	Treatment Length
**Gosman-Hedström 1998 [23]**	I1	104	46/58	Female: 75 No SD reported	NR	Ten acupuncture points according to traditional Chinese medicine were used on both paretic and the nonparetic sides. The needles on the nonparetic side were stimulated manually. Electrical stimulation was applied to the needles on the paretic side.	Two 30-minute sessions per week	10 weeks
				Male: 77 No SD reported				
	I2			Female: 82 No SD reported		Four short needles (15 mm) were used, 1 in each extremity. The needles were placed superficially and left for 30 minutes. No electrical or manual stimulation was applied.	Two 30-minute sessions per week	10 weeks
				Male: 76 No SD reported				
	C			Female: 78 No SD reported		Usual care	NR	10 weeks
				Male: 74 No SD reported				
**Hopwood 2008 [24]**	I	105	45/60	70.5 (42–93)*	NR	Electroacupuncture treatment was conducted. Needle points were based on all available best practice guidelines and used with a current of 2 Hz.	Three 30-minute sessions per week	4 weeks
	C			74.4 (61–93)*		A placebo intervention where body and scalp points were attached to TENS machine with red flashing lights and deactivated leads so that no current could flow.	Three 20-minute sessions per week	4 weeks
**Hsieh 2007 [25]**	I	63	35/28	68.8 No SD reported	NR	Electroacupuncture was performed according to traditional Chinese medicine. Nineteen acupoints were selected and used with alternating stimulation pulses (3 and 15 Hz).	Two 20-minute sessions per week, with a total of 8 sessions in one month.	4 weeks
	C			70.7 No SD reported		Usual care	NR	4 weeks
**Johansson 1993 [26]**	I	78	42/36	76 No SD reported	NR	Acupuncture was given on paretic and non-paretic sides using traditional Chinese acupuncture points with 10 needles, 4 of which provided electrical stimulation on paretic side. Also received usual rehabilitation care including daily PT and OT.	Two 30-minute sessions per week	10 weeks
	C			75 No SD reported		Usual care including daily PT and OT.	NR	NR
**Liu 2016 [22]**	I	38	14/4	65.6 (12.4)	14.2 (19.2) hours	Manual acupuncture using 3 needling techniques and 7 scalp points.	One 15–20 minute session daily.	2 weeks
	C		10/10	68.1 (9.16)	14.9 (18.1) hours	Usual care only	NR	NR
**Min 2008 [27]**	I	60	32/28	66.24 (10.2)	Haem: 7.8 (1.5) days	Usual rehabilitation plus acupuncture, delivered using needle points according to stages of Brunstrom criteria.	Five 30-minute sessions per week	NR
	C			65.78 (8.46)		Usual care	NR	NR
**Park 2005 [28]**	I	116	60/56	74.8 (10)	NR	Manually stimulated acupuncture using standard needles at recognised points based on Korean medicine. 10 needle points used, with 6 tailored to participant and 4 standard for stroke. Participants also received routine rehabilitation care.	Nine to twelve 20-minute sessions	2 weeks
	C			74.1 (10.2)		Sham treatment using non-penetrating needles 1.5cm away from recognised points. Participants also received routine rehabilitation care.	Nine to twelve 20-minute sessions	2 weeks
**Pei 2001 [29]**	I	86	52/34	71.61 (10.14)	3.32 (2.47) days	Usual care plus electro acupuncture.	Five 20-minute sessions per week	4 weeks
	C			69.34 (12.06)		Usual care.	NR	4 weeks
**Schuler 2005 [21]**	I1	120	NR	77.5 (7.4)	NR	Needling of acupuncture points with electrical stimulation.	Two 30-minute sessions per week	4 weeks
	I2			78.3 (5.9)		Sham intervention using surface electrodes on acupuncture points with visual stimulation	Two 30-minute sessions per week	4 weeks
	C			78.7 (7.4)		No intervention	NR	NR
**Sze 2002 [30]**	I	106	56/50	Mild: 69.3 (9.6)	NR	Participants received the same standard treatment as described for the control arm, together with traditional Chinese acupuncture.	Five 30-minute sessions per week, reducing to three 30-minute sessions per week post-discharge.	10 weeks
				Severe: 69.7 (11)				
	C			Mild: 71.9 (7.5)		Participants received standard treatment which included PT, OT, and speech therapy.	NR	10 weeks
				Severe:72.5 (6.8)				
**Zhu 2013 [20]**	I	188	130/ 58	65.51 (9.71)	14.4 (7.8) days	Acupuncture with needle points individualised to participant needs, and usual rehabilitation.	Weeks 1–4: 5 sessions per week Weeks 5–12: 2–3 sessions per week	12 weeks
	C			66.27 (11.16)		Usual rehabilitation.	NR	12 weeks

*Median and IQR reported by study

Haem. = Haemorrhagic stroke Infarct. = Stroke caused by infarction I1: Intervention arm 1 I2: Intervention arm 2 NR: Not Reported SD: Standard Deviation

#### Interventions

Interventions varied in their design (for example number of points used or whether manual or electrical stimulation was applied) and in their duration. Intervention descriptions are summarised in Table 2.

#### Risk of Bias

Eight (72.7%) studies were at risk of bias arising from unblinded or inadequately blinded participants. Many of the studies were unclear regarding their methods of randomisation and allocation concealment.

#### What is the effectiveness of acupuncture (traditional or electro) upon older stroke survivors ADL recovery in comparison to either usual rehabilitation care without acupuncture or sham treatment?

Of the 11 trials investigating the impact of acupuncture upon older stroke survivors ADL recovery (see Table 3), three studies demonstrated statistically significant benefit upon ADL scores favouring acupuncture [25–27]. Pei *et*.*al*. (2001) also found limited short term support for the use of acupuncture for ADL recovery, but any significant benefit had disappeared by week 2 post-intervention [29].

**Table 3:** Results of studies investigating the impact of acupuncture upon older stroke survivors ADL recovery.

Study	Intervention	Control	p-value	GRADE Score	GRADE Comment
**Gosman- Hedström 1998 [23]**	*Acupuncture*	*Control*	*BI Mean difference (95%CI)*:	Low	a. Eight studies involved unblinded participants and there were several uncertainties surrounding methods of randomisation and allocation. b. The variation in intervention design, delivery and duration suggests high heterogeneity between trials. c. Three of the eleven studies reported significant results favouring the acupuncture intervention.
	*BI (Mean*, *SD)*	BI (Mean, SD)			
	Base: 24 (SD NR)	Base: 26 (SD NR)	Base: NR		
	3mths: 38.18 (24.77)	3mths: 32.0 (27.34)	3mths: 2.1 (8.4, 12.6)		
	12mths: 41.94 (25.78)	12mths: 37.17 (26.28)	12mths: 1.65 (29.6, 12.9)		
	*Sham*		All p values NS		
	*BI (Mean*, *SD)*				
	Base: 26 (SD NR)				
	3mths: 32.0 (27.34)				
	12mths: 37.17 (26.28)				
**Hopwood 2008 [24]**	Base: 5.9 (3.97)	*BI (Mean*, *SD)*	*BI*		
	3wks: 9.6 (5.58)	Base: 6.9 (3.98)	*Base*: *NR*		
	6wks: 11.7 (5.61)	3wks: 10.1 (6.01)	*3wks*: *NR*		
	12wks: 12.9 (5.51)	6wks: 12.1 (5.81)	*6wks*: *NR*		
	24wks: 15.3 (4.72)	12wks: 13.3 (5.63)	12wks p = .737		
	52wks: 16.3 (4.3)	24wks: 15.9 (5.1)	*24wks*: *NR*		
	Base: 5.9 (3.97)	52wks: 15.2 (5.48)	52wks p = .371		
**Hsieh 2007 [25]**	*FIM (Mean change*, *SD)*	*FIM (Mean change*, *SD)*	*FIM*		
	Base: 59.0 (27.4)	Base: 60.7 (27.4)	Base: p = .78		
	2wks: 15.0 (9.8)	2wks: 10.8 (8.6)	2wks: p = .09		
	4wks: 25.2 (12.9)	4wks: 19.7 (15.9)	4wks: p = .18		
	3mths: 37.6 (18.9)	3mths: 27.4 (22.1)	3mths: p = .10		
	6mths: 44 (19.1)	6mths: 30.5 (24.5)	6mths: p = .05, ANOVA (Adjusted for group and time) p = .021 (without ITT) and p = .148 (with ITT)		
**Johansson 1993 [26]**	*BI (Mean*, *SD)*	*BI (Mean*, *SD)*	*BI*		
	Base: 45.1 (2.5)	Base: 45.1 (3)	Baseline: p = NR		
	1mth: 69.4 (3)	1mth: 60.6 (3.4)	1mth: p = < .05		
	3mths: 90.4 (2.2)	3mths: 72.4 (3.2)	3mths: p = < .0001		
	12mths: 92 (2.9)	12mths: 71.3 (4)	12mths: p = < .0001		
**Liu 2016 [22]**	*FIM (Mean change*, *SD)*	*FIM (Mean change*, *SD)*	*FIM*		
	1wks: 6.24 (4.82)	1wks: 6.06 (5.94)	1wks: p = .92		
	2wks: 11.12 (12.24)	2wks: 8.18 (7.58)	2wks p = .41		
	3wks: 12.12 (12.43)	3wks: 9.56 (7.72)	3wks: p = .48		
	4wks: 15.40 (13.40)	4wks: 10.13 (8.40)	4wks: p = .21		
**Min 2008 [27]**	*BI (Mean*, *SD*)	*BI (Mean*, *SD)*	*BI*		
	Base: 27.28 (5.41)	Base: 28.01 (4.48)	Base: p = NS		
	3mths: 80.78 (12.79)	3mths: 60.08 (11.92)	3mths: p = < .05		
**Park 2005 [28]**	*BI*	*BI*	*BI*		
	Base: 6 (3.8–9)	Base: 6 (4–9.3)	2wks p = NS		
	2wks: 11 (5–17)	2wks: 11 (7.3–15.8)	Mean change p = NS		
**Pei 2001 [29]**	*BI (Mean*, *SD)*	*BI (Mean*, *SD)*	*BI*		
	Base:25.1 (2.47)	Base: 24.3 (2.71)	Base: NR		
	1wks: 36.3 (3.41)	1wks: 28.2 (2.54)	1wk: p < .05		
	2wks: 46.7 (3.82)	2wks: 34.8 (2.64)	2wks: p < .01		
	4wks: 67.2 (4.51)	4wks: 41.6 (3.57)	4wks: p < .01		
	3mths: 82.9 (3.77)	3mths: 60.4 (3.26)	3mths: p < .01		
**Schuler 2005 [21]**	*Acupuncture*	*Control*	*BI*		
	*BI (Mean*, *SD)*	BI (Mean, SD)	Base: p = .99		
	Base: 37.1 (19.8)	Base: 38 (10.2)	4wks: p = .87		
	4wks: 50.7 (27)	4wks: 52.3 (24.6)	6mths: p = .69		
	6mths: 51.6 (40.9)	6mths: 44.7 (33.6)	*BI*		
	*Sham*				
	BI (Mean, SD)				
	Base: 37.4 (19.3)				
	4wks: 54 (25.8)				
	6mths: 50.4 (34.3)				
**Sze 2002 [30]**	*BI (Median*, *IQR)*	*BI (Median*, *IQR)*	*BI*		
	Base:	Base:	*Base*: *NR*		
	Severe: 7.2 (5.0–9.0)	Severe: 6.2 (3.7–9.0)			
	Moderate: 12.0 (11.0–13.0)	Moderate: 12.0 (11.0–13.0)			
	5weeks:	5weeks:	*5weeks*: *NR*		
	Severe: 14.8 (9.0–16.0)	Severe: 12.8 (8.1–17.0)			
	Moderate: 18.0 (16.0–19.4)	Moderate: 18.0 (17.0–19.4)			
	10 weeks:	10 weeks:	*10weeks*:		
	Severe: 16.2 (10.1–18.0)	Severe: 14.7 (8.7–18.0)	Severe: p = .596		
	Moderate: 19.0 (15.9–20.0)	Moderate: 19.0 (18.0–20.0)	Moderate: p = .469		
**Zhu 2013 [20]**	*BI (Mean*, *SD)*	*BI (Mean*, *SD)*	*BI* Mean difference (95% CI):		
	Base: 31.07 (16.84)	Base: 33.82 (18.79)	*Base*: *NR*		
	1mth: 54.03 (20.83)	1mth: 59.72 (27.4)	*1mts*: *NR*		
	2mths: 72.65 (20.98)	2mths: 74.03 (20.94)	*2mths*: *NR*		
	3mths: 80.36 (21.07)	3mths: 82.66 (18.94)	*3mths*: 2.99 (2.45, 8.44) p = .282		

Base: Baseline BI: Barthel Index FIM: Functional Independence Measure IQR: Inter-quartile Range ITT: Intention to Treat analysis Mths: Months NR: Not Reported NS: Not Significant SD: Standard Deviation Wks: Weeks

It should be noted that those reporting significant findings in relation to ADL recovery following acupuncture tended to be smaller trials. Quality assessment, using the GRADE approach identified the overall body of to be low (see Table 3) due to risks imposed by various biases and the heterogeneity between intervention content, delivery and duration. This means that further studies are very likely to have an important impact on the estimate of effect. Overall, the evidence does not support the use of acupuncture after stroke.

#### What is the effectiveness of acupuncture upon older stroke survivors’ disability recovery in comparison to usual rehabilitation care without acupuncture or sham treatment?

Liu et al (2016) was the only trial to assess the effect of acupuncture upon stroke survivors’ disability recovery [22]. In their small trial (n = 38) no significant differences (p = .15) in mean modified Rankin scale scores were identified between those receiving acupuncture (1.13, SD 1.25) and those receiving usual care only (1.27, SD 1.16) at 12 weeks post-intervention. A GRADE assessment found the evidence to be moderate but due to the limited number of studies, and the small sample size, further studies are likely to impact upon the expected effect identified in this review. We therefore cannot recommend acupuncture to improve older stroke survivors post-stroke disability.

### Caregiver training

#### Studies

Only one study investigated the effect of caregiver training upon older stroke survivors ADL recovery. The RCT [31] was conducted within an inpatient rehabilitation unit in one UK hospital.

#### Participants

Of 300 participants, 53% (n = 160) were male. The intervention group had a median age of 76 years (IQR 70–80) versus the control group median of 76 years (IQR 70–82). Time between stroke onset and intervention was not reported.

#### Intervention

Caregiver training consisted of three to five 30–45 minute sessions of instruction. Sessions covered common stroke related problems, their prevention and management, and included hands on training in moving and handling, mobility encouragement, transfers, and speech/ communication. Sessions were conducted in the hospital whilst the participant was an inpatient. One final session was delivered to the caregiver in the participant’s home environment following participants discharge. The control group participants received usual care only.

#### Risk of bias

A lack of blinding represents the most significant risk of bias for this study

#### Can pre-discharge caregiver training influence post-discharge stroke survivor ADL recovery in comparison to those who receive no caregiver training?

One study, by Kalra *et*. *al*. *(*2004), investigated if caregiver training could influence ADL recovery [31]. Twelve months post intervention, the intervention group scored a median Frenchay Activities Index (FAI) score of 15 (IQR 9–23) versus the control participants median of 16 (IQR 8–22). The difference between groups on this measure was not significant. Using the Barthel Index (BI), presented as the number of persons considered 'improved', classified by BI score >18 at the measurement point, 51% (n = 77) of the intervention participants at three months were considered ‘improved’ versus 35% (n = 52) of the control participants. The difference in the number of persons ‘improved’ was found to be significant (p = .007). At 12 months, 62% (n = 93) of the intervention participants were considered improved, versus 50% (n = 75) of the control participants. However, the difference between groups at 12 months was not significant (p = .074); improvement in ADL recovery was not sustained over time. Pre-discharge caregiver training may offer some benefit to older stroke survivors ADL recovery in the short term. However, only one study investigated this type of intervention and actual BI scores were not presented. GRADE quality assessment was low. Insufficient evidence means we cannot make a recommendation for this type of intervention. There is, however, evidence warrants further investigation as to the potential benefits of such an intervention.

#### Can pre-discharge caregiver training effect post-discharge stroke survivor Disability scores in comparison to those who receive routine post-discharge advice with no caregiver training?

Only one study [31] investigated if caregiver training could influence participant disability scores. Disability was assessed with the modified Rankin score, presented as the number of persons considered 'improved' as classified by a Rankin score of 0–2. At three months, 53% (n = 80) of intervention participants, versus 42% (n = 63) of control participants, were considered improved. At 12 months, 66% (n = 100) of intervention participants, versus 58% (n = 87) of control participants, were considered improved. However, differences between groups were not significant at either time point (p = .054, p = .18). Very limited evidence from one RCT, with a GRADE assessment of low, suggests no benefit to older stroke survivors’ disability arising from caregiver training.

### Constraint induced movement therapy

#### Studies

Two RCTs exploring the benefits of Constraint induced movement therapy (CIMT) upon older stroke survivors were identified [32–33]. Both were conducted using rehabilitative outpatient departments, but one was conducted in Taiwan [33] and the other in China [32].

#### Participants

The trials involved a total of 116 participants, 56% (n = 65) of whom were male. Mean age ranged from 65.07 (SD 6.7) years [32] to 71.94 (SD 6.79) years [33]. The mean time since stroke ranged from 8.44 (SD 0.62) days [32] to 7.5 months [33].

#### Interventions

Wu *et*. *al*. (2007) employed a modified CIMT technique (n = 13) where subjects placed unaffected hands in self-adhesive strapping for six hours per day while at home [33]. In addition, participants received two-hour sessions of CIMT with a therapist for five days per week. Sessions, provided after participants’ regular OT appointments, focused on carrying out basic ADLs with the affected arm while the unaffected hand was constrained. This was compared against a traditional rehabilitation programme (n = 13) that focussed on practising ADLs but without constraining the affected limb.

Liu *et*. *al*. (2016) compared self-regulated modified CIMT (n = 30), modified CIMT (n = 30), and a control group (n = 30) employing conventional rehabilitation [32]. Self-regulated CIMT participants were encouraged to reflect on their abilities and come up with their own solutions to problems, rather than follow instructions. Modified CIMT participants received therapist feedback and improvement suggestions during arm restraint training sessions. Each group practised 10 tasks daily, through graded and progressive exercises, over two weeks, for one hour per day, five days per week.

#### Risk of bias

Risk of bias from both studies is possible due to unblinded participants and lack of clarity surrounding allocation concealment.

#### What is the effectiveness of CIMT upon older stroke survivors ADL recovery in comparison to those receiving conventional rehabilitation only?

Wu *et*. *al*. (2007) reports there to have been no significant difference between the groups (p = .018) at 3 weeks follow-up [33]. Lui *et*. *al*. (2016) report that participants receiving self-regulated modified CIMT had significantly higher Instrumental Activities of Daily Living (IADL) scores at week 2 (p < .001) than those receiving modified CIMT alone or conventional therapy [32]. However, this difference had disappeared at 1 month follow up (p = .51). The limited number of studies, combined with a low GRADE quality assessment means that the evidence base is inadequate to conclude CIMT is effective for older stroke survivors ADL.

### Device assisted physiotherapy

#### Studies

Eight RCTs exploring device assisted physiotherapy were identified. Two each were conducted in France and Italy, and one each from the USA, China, Sweden and the UK.

#### Participants

In total 422 participants were randomised, with mean ages ranging from 65 years [34] to 75 years [35]. Table 4 summarises participant characteristics.

**Table 4:** Participant characteristics and study descriptions of included device assisted physiotherapy interventions.

Study	Arm	No. of Participants	Male/ Female	Age (Yrs, Mean, SD)	Time post-stroke (Mean, SD)	Description	Timing	Treatment Length
**Bagley 2005 [36]**	I	141	55/ 86	75.8 (11.5)	19.5 (12.1) days	Intervention participants were expected to receive 14 consecutive daily treatments using the Oswestry Standing Frame.	1 session per day, up to 14 sessions	2 weeks
	C			75.1 (9.4)		Control participants were expected to receive 14 consecutive treatments without access to the Oswestry frame.	1 session per day, up to 14 sessions	2 weeks
**de Sèze 2001 [34]**	I	20	11 / 9	63.5 (17)	36.8 (25) days	Participants followed a program using the Bon Saint Come device for axial postural rehabilitation, a technique based on voluntary trunk control during exploratory retraining. Participants also received usual rehabilitation.	Month 1: One 1-hour session using the device Months 2&3: One 2-hour conventional neurorehabilitation session daily.	3 months
	C			67.7 (15)		Control participants had conventional neurorehabilitation only.	One 2-hour session of conventional neurorehabilitation each day.	3 months
**Franceschini 2009 [37]**	I	102	50 / 47	65.5 (12.2	28.9 (12) days	Participants completed gait training on a treadmill with body weight support followed by conventional training.	One 20-minute session per day, 5 days per week, up to 20 sessions.	5 weeks
	C			70.9 (11.8)		Participants completed conventional over ground gait training.	One 60-minute session per day, 5 days per week, up to 20 sessions.	5 weeks
**Masiero 2007 [38]**	I	35	21 / 14	63.4 (11.8)	NR	Experimental participants received, in addition to usual care, early sensori-motor robotic training by NeReBot using their impaired upper limb.	One session per day, five days per week	5 weeks
	C			68.8 (10.5)		The control group received initial exposure to the robot but exercises were performed with the unimpaired upper limb.	One session per day, five days per week	5 weeks
**Ng 2008 [39]**	I1	54	34 / 19	66.6 (11.3)	2.7 (1.2) weeks	Participants in the Gait Trainer group trained on the electromechanical gait trainer with their body weight partially supported.	20-minute sessions (frequency and no. of sessions NR)	4 weeks
	I2			62 (10.0)		Subjects in the Gait Trainer and Functional Electrical Stimulation group underwent the same training on the gait trainer as the Gait Trainer group but also received Functional Electrical Stimulation simultaneously.	20-minute sessions (frequency and no. of sessions NR)	4 weeks
	C			73.4 (11.5)		Control group participants received conventional physiotherapy gait training.	NR	4 weeks
**Rabadi 2008 [40]**	I1	30	19 / 11	69.2 (10.22)	22.2 (15.11) days	The Monark arm ergometer (Monark-Crescent AB of Sweden) is a bidirectional hand cycle. The paretic arm was supported by a wrist splint and then placed on the arm ergometry arm pedal. The subject exercised for 20 minutes of continuous cycling, had a 5-minute rest, and then cycled again for another 20 minutes.	One 40-minute session per day, 5 days per week. Received up to 12 sessions.	NR
	I2			79.5 (6.7)		Robot-aided therapy used a robot (MIT- Manus) which consists of goal-directed, robot-assisted arm movement. A customized interactive computer-generated video programme provided visual feedback to the participant about the speed and accuracy of reaching the target.	One 40-minute session per day, 5 days per week. Received up to 12 sessions.	NR
	C			67.8 (12.66)		The control occupational therapy group received group therapy led by a certified occupational therapist.	One 40-minute session per day, 5 days per week, up to 12 sessions.	NR
**Rydwik 2006 [35]**	I	18	13 / 5	74.9 (8.7)	48.7 (19.6) months	Stimulo (Farzaneh Chidopory, Sweden) is a portable device developed to maintain or increase range of motion in the ankle. The intervention was standardized, and a warm up was followed by a period of 15–20 min of active and passive exercises individualized by muscle strength in the ankle. The subjects were instructed to hold for 10 s in maximum range of motion positions.	Three 30-minute sessions per week.	6 weeks
	C			75.3 (4.9)		NR	NR	NR
**Wiart 1997 [41]**	I	22	12 / 10	66 (8)	35 (9) days	Participants received treatment using the Bon Saint Come’s device, where the participant is forced to make an axial rotation of the trunk under visual control. Participants also received traditional rehabilitation.	One 60-minute session daily using the device, and 2–3 hours conventional therapy daily.	20 days
	C			72 (6)		Control participants received 3 to 4 hours of traditional rehabilitation each day.	3–4 hours daily.	20 days

*Median and IQR given

C: Control I: Intervention I1: Intervention arm 1 I2: Intervention arm 2 NR: Not Reported SD: Standard Deviation

#### Interventions

Interventions varied in their content and duration and are described in Table 4. We subdivided trials into those using either robotic or non-robotic devices.

#### Risk of bias

All studies were at risk of bias resulting from unblinded or inadequately blinded participants, although most had blinded assessors.

#### What is the effectiveness of a robotic physiotherapy device upon older stroke survivors ADL recovery in comparison to those receiving conventional physiotherapy?

Two studies [38,40] assessed effectiveness of robot-assisted therapy versus usual care (presented in Table 5). Maseiro *et*. *al*. (2007) compared a sensorimotor robotic training programme against usual care [38]. Although intervention participants had a significantly better Functional Independence Measure (FIM) score at 6 weeks than control participants, its benefit was not sustained at three or eight month follow-up. The findings of Rabadi *et*. *al*. (2008), in their comparison of arm ergometer training, the MIT-MANUS robotic trainer, and usual care, also suggest that additional physiotherapy devices offered no further benefit than usual rehabilitative care [40].

**Table 5:** Results of studies investigating the impact of robotic device physiotherapy interventions upon older stroke survivors ADL recovery.

Study	Intervention	Control	p-value	GRADE Score	GRADE Comment
**Maseiro 2007 [38]**	*FIM (Mean*, *SD)*	*FIM (Mean*, *SD)*	*FIM (Mean*, *SD)*	Very Low	a. High risk of bias across all included studies b. Substantial heterogeneity c. Total sample size <400 d. One of two studies reported that robot assisted physiotherapy approaches can significantly benefit ADL recovery.
	6wks: 32.6 (7.2)	6wks: 25.5 (10.5)	6wks: p < .05		
	3mths: 44.2 (12.1)	3mths: 29.7 (14.5)	3mths: p < .01		
	8mths: 46.2 (10.4)	8mths: 31.8 (14.6)	8mths: p < .01		
**Rabadi 2008 [40]**	*Ergometer*	*Control*	*FIM*		
	*FIM (Mean*, *SD)*	*FIM (Mean*, *SD)*			
	6wks: 68.42 (5.24)	6wks: 70.76 (7.1)	6wks: p = NS		
	*Robot*				
	*FIM (Mean*, *SD)*				
	6wks: 60.09 (6.42)				

ADL: Activities of Daily Living FIM: Functional Independence Measure Mths: Months NS: Not Significant SD: Standard Deviation Wks: Weeks

The substantial heterogeneity, combined with a serious risk of bias due to lack of methodological reporting, resulted in a downgrading of the quality of the evidence to very low (see Table 5).

#### What is the effectiveness of a non- robotic physiotherapy device upon older stroke survivors ADL recovery in comparison to those receiving conventional physiotherapy?

Six studies investigated the impact of non-robotic physiotherapy devices upon stroke survivors ADL recovery (presented in Table 6). Only one of the six studies, Wiart *et*. *al*. (1997), reported a moderate significant benefit favouring the non-robotic device at day 30, versus usual care control (p < .03) [41]. That said, there appears to be an imbalance between baseline scores of the two groups and there is no report if this was significant, nor any reporting of the difference between the change in means between groups. The quality of evidence of these studies was rated as very low (see Table 6), owing to a serious risk of bias and small sample size.

**Table 6:** Results of studies investigating the impact of non-robotic device physiotherapy interventions upon older stroke survivors ADL recovery.

Study	Intervention	Control	p-value	GRADE Score	GRADE Comment
**Bagley 2005 [36]**	*BI (Mean change*, *SD)*	*BI (Mean change*, *SD)*	*BI*	Very Low	a. All studies judged to be at high risk of bias b. Lack of optimal information size (total participants <400) c. One of six studies reported significant benefit upon ADL from use of a non-robotic device
	Base: 1(0–3) *	Base: 2 (1–3) *	All p = NS		
	6wks: 3.2 (4.3)	6wks: 2.89 (3.6)			
	12wks: 4.66 (4.9)	12wks: 4.76 (4.6)			
	6mths: 5.44 (5.9)	6mths: 6.2 (5.2)			
**De Seze 2001 [34]**	*FIM (Mean*, *SD)*	*FIM (Mean*, *SD)*	*FIM*		
	Base: 71.0 (16.9)	Base: 79.6 (14.9)	All p = NS		
	Day 30: 99.4 (10.8)	Day 30: 101.7 (14.3)			
	Day 90: 109.6 (10.5)	Day 90: 110.0 (12.8)			
**Francheschini 2009 [37]**	*BI (Median*, *IQR)*	*BI (Median*, *IQR)*	*BI*		
	Base: 6 (3–9)	Base: 5 (3–7)	All p = NS		
	2wks: 15 (11.8–18)	2wks: 15 (11–18)			
	6mths: 17 (14.5–18.5)	6mths: 17.5 (14–19)			
**Ng 2008 [39]**	*Gait Training*	*Control*	*BI*		
	*BI (Mean*, *SD)*	*BI (Mean*, *SD)*	All p = NS		
	Base: 54.4 (13.5)	Base: 53.3 (12.4)			
	4wks: 79.1 (9.4)	4wks: 76.3 (19.3)			
	6mths: 83.8 (19.3)	6mths: 80.8 (21.4)			
	*FIM (Mean*, *SD)*FIM	*FIM (Mean*, *SD)*FIM (Mean, SD)	*FIM*		
	Base: 78.6 (12)	Base: 78.6 (8.9)	*All p = NS*		
	4wks: 103.2 (17.6)	4wks: 98.2 (14.3)			
	6mths: 107.2 (15.1)	6mths: 102.5 (16.5)			
	*Gait Training & FES*				
	*BI (Mean*, *SD)*				
	Base: 46.4 (13.6)				
	4wks: 73.6 (19)				
	6mths: 80.7 (14.9)				
	*FIM (Mean*, *SD)*				
	Base: 65 (16.8)				
	4wks: 86.4 (20.5)				
	6mths: 94 (20.2)				
**Rydwik 2006 [35]**	*ADL (Median*, *IQR)*	*ADL (Median*, *IQR)*	*ADL*		
	Base: 109 (100–115)	Base: 107 (113–116)	All p = NS		
	6wks: 107 (99–113)	6wks: 112 (105–114)			
	*IADL (Median*, *IQR)*	*IADL (Median*, *IQR)*	*IADL*		
	Base: 20 (14–36)	Base: 26 (20–35)	All p = NS		
	6wks: 21 (18–39)	6wks: 28 (15–40)			
**Wiart 1997 [41]**	FIM (Mean, SD)	FIM (Mean, SD)	*FIM*		
	Base: 66 (17)	Base: 54 (10)	*Base*: *NR*		
	Day 30: 86 (23)	Day 30: 62 (14)	Day 30: P < .03		

*Baseline given as median/ IQR, f/up as mean change

ADL: Activities of Daily Living Base: Baseline BI: Barthel Index FES: Functional Electrical Stimulation FIM: Functional Independence Measure IADL: Instrumental Activities of Daily Living IQR: Inter-quartile Range Mths: Months NS: Not significant SD: Standard Deviation Wks: Weeks

### Music therapy

#### Studies

One RCT, conducted in Italy, investigated the role of music therapy in the treatment of older stroke survivors ADL.

#### Participants

The study [42] involved 38 participants, 42.1%(n = 16) of whom were male. The mean age of participants in the intervention arm was 70.4 (SD 8.9) years versus 75.4 (SD 7.6) of control participants. All participants commended intervention within six to eight weeks of stroke onset.

#### Intervention

Intervention participants received Relational Active Music Therapy, conducted by trained musical therapists. Participants were encouraged to use rhythmical instruments during these sessions, which were provided three times per week and lasted for around 30 minutes per session. Participants received up to 20 sessions in total. Control participants received no additional intervention.

#### Risk of bias

For most bias types, this study was rated as being unclear due to insufficient reporting

#### Can music therapy effect stroke survivors ADL recovery against usual care alone?

Both intervention and control participants in this small study improved over time (p < .001) but no significant difference between groups final scores or change in scores from baseline were identified [42]. Music therapy participants improved their mean FIM scores from 76.58 (20.35) at baseline, to 110.47 (9.9) at follow up. Similarly, control participants improved their FIM scores from 71.26 (19.33) to 106.89 (16.83). The inclusion of only one small study, with an unclear risk of bias, which demonstrated no improvement in ADL, means that we are not able to recommend the use of music therapy to improve ADL recovery amongst older stroke survivors. The evidence has been given a GRADE quality assessment of low, meaning that further studies are very likely to change the effect estimate.

### Nerve stimulation

#### Studies

Three RCTs presented findings in relation to nerve stimulation devices and ADL recovery amongst older stroke survivors. Two studies were undertaken in Sweden, and one study undertaken in the United States of America (USA).

#### Participants

In total, 232 participants were randomised, of which 62.5% (n = 145) were male. Table 7 presents a summary of participant characteristics.

**Table 7:** Participant characteristics and study descriptions of included nerve stimulation interventions.

Study	Arm	No. of Participants	Male/ Female	Age (Yrs, Mean, SD)	Time post-stroke (Mean, SD)	Description	Timing	Treatment Length
**Johansson 2001 [43]**	I1	150	90/60	76 (9)	NR	Acupuncture treatment alternating between 2 modes (9 and 10 needle points) with low frequency electro stimulus.	Thirty-minute session, twice per week	10 weeks
	I2			77 (9)		Trans electrical nerve stimulation (TENS) treatment with high intensity low frequency electrodes used in same areas as acupuncture points.	Thirty-minute session, twice per week	10 weeks
	C			76 (11)		Sham treatment using the same equipment and electrode placement as TENS intervention, but with low intensity.	Thirty-minute session, twice per week	10 weeks
**MacDonell 1994 [44]**	I	38	28/10	65 (9)	25 days (11–41) *	Cyclical electrical stimulus (CES) was conducted upon the Common Peroneal Nerve at the knee joint. Additionally, Functional Electrical Stimulation (FES) was used whilst participant was performing exercises/ activities which are graded to the individuals’ abilities.	CES: 30 to 40-minute sessions, 5 sessions per week FES: 20-minute sessions, three sessions per week	4 weeks
	C			68 (9)		Participants worked through a self-exercise program to develop muscle strength. To replace FES, participants performed exercise/activity sessions involving the same tasks as used in the FES intervention, but without electrical stimulation.	Exercise: 20-minute sessions, three times per week. PT: 15-minute sessions, two to three times daily.	4 weeks
**Sonde 1998/2000 [45–46]**	I	44	27/17	71 (6)	9.1 months (2.2)	Low frequency TENS with electrodes placed at wrist extensors of effected arm. In 80% of intervention participants, electrodes were also placed at elbow extensors and shoulder abductors. First 3 sessions delivered by physiotherapist, thereafter TENS was applied by participant at home.	One-hour session per day, 5 sessions per week.	3 months
	C			73 (3.5)		Usual care.	NR	3 months

*Median and IQR given

C: Control I: Intervention I1: Intervention arm 1 I2: Intervention arm 2

#### Interventions

The three studies varied in their type of nerve stimulation, location of bodily impairment targeted and duration of treatment. Table 7 presents a summary of each intervention.

#### Risk of bias

Of the three studies, two studies had a lack of or inadequate blinding procedures. Insufficient reporting to clarify risk of several other bias sources resulted in a number of bias assessments being unclear.

#### Can the use of nerve stimulation devices influence older stroke survivors ADL recovery in comparison to those who receive usual rehabilitation care or sham treatment only?

Three studies explored the efficacy of nerve stimulation device use upon older stroke survivors ADL recovery (see Table 8 for study results). Johansson *et*. *al*. (2001) and MacDonell *et*. *al*. (1994), who both studies participants in the acute phase of stroke recovery, found no significant difference in BI scores between those receiving nerve stimulation and those receiving usual care [43–44]. Conversely, Sonde *et*. *al*. (1998; 2000), in their 3-year follow up study involving chronic post-stroke survivors, suggests that Trans-electrical Nerve Stimulation (TENS) may not result in significant improvements in ADL immediately following intervention but may allow older stroke survivors to better maintain ADL scores in the 3 years following stroke [45–46]. While both the intervention and control groups ADL scores declined from 3 months to the 3 year follow up, the control groups reduction in ADL was significantly greater than those who received the TENS intervention [45–46].

**Table 8:** Results of studies investigating the impact of nerve stimulation interventions upon older stroke survivors ADL recovery.

Study	Intervention	Control	p-value	GRADE Score	GRADE Comment
Johansson 2001 [43]	*BI*	*BI*	*BI*	Very Low	a. Several sources of bias including unblinded patients b. Studies differ in terms of acute v chronic stroke survivors, intervention type (TENS, FES), and duration of intervention (range 4wk-3mths). c. Small sample size d. 2 of the three studies reported no significant results in favour of nerve stimulation, however the results of one study suggests nerve stimulation may offer protective benefits over any future ADL decline.
	NR	NR	p = NS		
MacDonell 1994 [44]	*BI (Mean*, *SD)*	*BI (Mean*, *SD)*	*BI*		
	Base: 9.3 (2.7)	Base: 8.9 (2.9)	Base: p = NS		
	4wks: 14.7 (2.6)	4wks: 12.9 (3.7)	4wks: p = NS		
	8wks: 17.6 (2.6)	8wks: 15.8 (3.3)	8wks: p = NS		
Sonde 1998/2000 [45–46]	*BI (Mean*, *SD)*	*BI (Mean*, *SD)*	*BI*		
	Base: 80 (13.5)	Base: 79.5 (10.7)	Base: p = NS		
	End of treatment: 81.9 (13.3)	End of treatment: 79.0 (10.7)	End of treatment: p = NS		
	3 years: 78.1 (16.6)	3 years: 66.5 (22.4)	3 years: ANOVA f = 3.6, p < .05		

Base: Baseline BI: Barthel Index NR: Not Reported NS: Not Significant SD: Standard Deviation Wks: Weeks

The evidence for the use of nerve stimulation for ADL recovery in older stroke survivors is limited (see Table 8). Nerve stimulation does not appear to improve ADL in the immediate term, but may offer protective benefits over future decline in ADL in the years following stroke. Quality assessment suggests the evidence to be very low which means that further studies will likely have an impact on findings. At present, it is not possible to recommend the use of nerve stimulation in the treatment of older stroke survivors to enhance ADL recovery.

### Occupational therapy

#### Studies

Twelve RCTs were included. Eight were conducted in the UK, with one each from China, Holland, Canada and Italy.

#### Participants

In total, 1632 participants were randomised, of which 49.6% (n = 810) were male. Mean ages ranged from 65.9 (SD 8.16) years [47] to 88.6 (SD 6.5) years [48]. Most studies did not report time between stroke onset and commencement of intervention. Those who did are presented in Table 9 alongside other participant characteristics.

**Table 9:** Participant characteristics and study descriptions of included occupational therapy interventions.

Study	Arm	No. of Participants	Male/ Female	Age (Yrs, Mean, SD)	Time post-stroke (Mean, SD)	Description	Timing	Treatment Length
**Chiu 2004 [49]**	I	53	35/18	72.1 (6.36)	NR	Assistive devices were demonstrated while participants were in hospital and intervention group participants received additional home-based training in the use of these devices by occupational therapists.	2–3 sessions	3 months
	C			72.2 (9.53)		The control group did not receive any treatment post-discharge.	NR	3 months
**Corr 1995 [50]**	I	110	41/ 69	75.1 (41–96) *	NR	Intervention involved teaching new skills; facilitating more independence in activities of daily living; facilitating return of function; enabling participants to use equipment supplied by other agencies; giving information to the participant and carer; and referring to or liaising with other agencies.	NR	NR
	C			75.8 (54–94)*		Usual care	NR	NR
**Donkervoort 2001 [51]**	I	113	64/ 49	67.6 (11.7)	100.2 (63.3) days	Strategy training consisted of the use of strategies to compensate for the apraxic impairment during the performance of activities in daily living.	Mean no. of sessions: 25 (SD 9.8) Mean treatment duration:15 hours (SD7.7)	8 weeks
	C			63.3 (11.6)		Usual occupational therapy concentrating on (sensory)motor, perceptual and cognitive deficits of the stroke participant.	Mean no. of sessions: 27 (SD 15.6) Mean treatment duration: 19 hours (SD 15.0)	8 weeks
**Gilbertson 2000 [52]**	I	138	60/ 78	71 (28–89)*	31 (17–57) days*	Client centred occupational therapy service involving 10 home visits over 6 weeks. Tailored to individual goals around self-care, and participation in domestic and leisure activities.	10 visits, 30–45 minutes per visit	6 weeks
	C			71 (31–89)*		Routine care	NR	NR
**Jongbloed 1989 [53]**	I	90	41/ 49	All: 71.32 (9.07)	40 (42) days	Functional occupational therapy: Emphasis is upon treating symptoms not the cause and involves practising tasks, usually in relation to activities of daily living, to increase independence.	40 minutes per day, 5 days per week	8 weeks
	C					Sensorimotor integrated occupational therapy: Emphasis is upon treating the cause not the symptoms and assumes a holistic approach whereby the motor and sensory systems are interdependent. It involves planned sensory inputs designed to improve motor skills.	40 minutes per day, 5 days per week	8 weeks
**Landi 2006 [54]**	I	50	23/ 27	78.3 (9.4)	NR	Combined occupational therapy and physiotherapy intervention (Physio is as per usual care on the ward). Occupational therapy programme individualised to individuals needs and involved practising personal care activities such as personal hygiene, feeding, toileting, dressing, mobility, to achieve and improve independence in these activities.	3 hours per day	8 weeks
	C			74.9 (10.9)		Usual care (without occupational therapy)	3 hours per day	8 weeks
**Logan 1997 [55]**	I	111	56 / 55	71 (10.2)	NR	An enhanced service where treatment was the same as 'usual' occupational therapy, but participants were seen quicker and more often.	Mean no. visits: 6 Mean minutes of therapy: 222 (SD 136)	6 months
	C			74 (11.5)		NR	Mean no. visits: 2.5 Mean minutes of therapy: 55 (SD 83)	6 months
**Logan 2004 [56]**	I	168	91/ 77	74 (8.4)	11 (8.4) months	An occupational therapist made a clinical assessment of the barriers to outdoor mobility, negotiated mobility goals, and then delivered interventions to achieve those goals.	Up to 7 sessions	3 months
	C			74 (8.6)		Leaflets describing local transport services for disabled people.	NR	3 months
**Parker 2001 [57]**	I1	466	269/ 197	72 (65–79)*	NR	The treatment goals in the leisure group were set in terms of leisure activity and so interventions included practising the leisure tasks as well as any ADL tasks necessary to achieve the leisure objective.	Minimum of 10 sessions, each lasting at least 30 minutes	NR
	I2			71 (66–78)*		The treatment goals in the ADL group were in terms of improving independence in self-care tasks and therefore treatment involved practising these tasks (such as preparing a meal or walking outdoors).	Minimum of 10 sessions, each at least 30 mins.	NR
	C			72 (65–78)*		Usual care	NR	NR
**Sackley 2006 [48]**	I	118	20/98	88.6 (6.5)	NR	Additional occupational therapy techniques to improve performance in activities of daily living	Median of 2.7 sessions per month per participant (IQR 1–4.2)	3 months
	C			86.3 (8.8)		Usual care	NR	3 months
**Walker 1996 [47]**	I	30	16 / 14	65.9 (8.16)	NR	Treatment was given by a senior occupational therapist at the participants home. Dressing practice was given on a regular basis, with the amount of therapy at the therapist’s discretion. Treatment involved teaching participants and carers appropriate techniques such as dressing the affected limb first, energy conservation, the use of red thread to overcome perceptual difficulties and to mark alignment of buttons, and advice on choice of clothing. Relatives were encouraged to continue the dressing practice between sessions with the occupational therapist.	NR	3 months
	C			70.2 (10.35)		No intervention	NR	3 months
**Walker 1999 [58]**		185	94/ 91	73.6 (8.1)	NR	Participants received visits from a research occupational therapist for up to 5 months. The frequency of treatment was agreed between the therapist, participant, and, if relevant, the carer. The aim of therapy was independence in personal and instrumental activities of daily living and the focus of therapy was active intervention rather than assessment or liaison.	Mean no. visits: 5·8 (SD 3·3) Mean length of each visit: 52 minutes (SD 11·8)	5 months
				75.1 (8.6)		No intervention	NR	NR

*Median and IQR given

C: Control I: Intervention I1: Intervention arm 1 I2: Intervention arm 2 IQR: Interquartile Range NR: Not Reported

#### Interventions

Interventions varied widely in their content and duration and are summarised in Table 9.

#### Risk of bias

Ten of the twelve studies involved unblinded or inadequately blinded participants, and two studies had inadequate assessor blinding.

#### Does increased occupational therapy intensity influence older stroke survivors ADL recovery against no occupational therapy or usual occupational therapy care?

As summarised (Table 10), only four of the ten RCTs reported any positive impact upon ADL scores from an occupational therapy (OT) intervention [49, 52, 54, 58]. In the study by Chiu *et*. *al*. (2004) both control and intervention participants mean FIM scores improved and the difference in mean change scores was significant (p = .001), favouring the additional occupational therapy [49]. However, no follow up beyond the end of intervention was conducted. Gilbertson *et*. *al*. (2000) found significant improvement in mean ADL scores between their control and intervention participants at eight weeks post-intervention, however these differences were not maintained at six months [52]. Conversely, Walker *et*. *al*. (1999) found significant improvement in ADL scores at six months post-intervention, favouring the home-based occupational therapy programme [58]. For the studies which were unable to demonstrate significant benefit, there was little, if any, change in ADL scores, regardless of outcome measure used.

**Table 10:** Results of studies investigating the impact of increased occupational therapy upon older stroke survivors ADL recovery.

Study	Intervention	Control	p-value	GRADE Score	GRADE Comment
**Chiu 2004 [49]**	*FIM (Mean*, *SD)*	*FIM (Mean*, *SD)*	*FIM (Mean*, *SD)*	Low	a. All included trials involved unblinded participants and a number of unclear bias risks were noted due to insufficient reporting b. Variation in the settings of trial interventions (e.g. inpatient rehab. units, nursing homes, participants own homes) and variation in the aims and content of interventions. Also substantial differences in duration of interventions, from 8 weeks to 6 months. c. A variety of measures were utilised to measure ADL across the studies, and four of ten studies reported significant results favouring an increased OT intervention.
	Base: 97.6 (10.7)	Base: 97.7 (11.8)	Base: NR		
	Post-test: 108.9 (11.6)	Post-test: 104.9 (12.0)	Post-test p = .001		
	Mean Difference: 11.4 (4.2)	Mean Difference: 7.0 (3.7)	Mean difference p = .001		
**Corr 1995 [50]**	*BI (Median*, *IQR)*	*BI (Median*, *IQR)*	*BI*		
	12mths: 13 (10–15)	12mths: 12 (6–15)	12mths p = NS		
	*NeADL (Median*, *IQR)*	*NeADL (Median*, *IQR)*	*NeADL*		
	12mths: 3 (0–20)	12mths: 2 (0–21)	p = NS		
**Gilbertson 2000 [52]**	*NeADL (Median*, *IQR)*	*NeADL (Median*, *IQR)*	*NeADL*: *Mean difference (95% CI)*:		
	Base: NR	Base: NR	*Base*: *NR*		
	8wks: 27 (19–43)	8wks: 23 (11–35)	8wks: 4 (-0.05, 10.0) p = 0.08		
	6mths: 28 (15–38)	6mths: 21 (14–38)	6mths: 7 (-3.6, 7.8) p = 0.48		
	*BI (Median*, *IQR)*	*BI (Median*, *IQR)*	*BI Mean difference (95% CI)*:		
	Base: 17 (15–18)	Base: 18 (16–19)	*Base*: *NR*		
	8wks: 18 (16–20)	8wks:17 (14–19)	8wks: 1 (0.0,2.3) p = .06		
	6mths: 17 (15–19)	6mths: 17 (13–18)	6mths: 0 (-0.6, 2.4) p = .25		
**Landi 2006 [54]**	*ADL (Mean*, *SD)*	*ADL (Mean*, *SD)*	*ADL*		
	Base:30.7 (6.1)	Base: 30.8 (7.8)	Base: p = .9		
	8wks: 13.2 (9.9)	8wks: 20.3 (11.5)	8wks p = .02		
**Logan 1997 [55]**	*BI (Median*, *IQR)*	*BI (Median*, *IQR)*	*BI*		
	3 mths: NR	3 mths: NR	p = NR		
	6 mths: 16 (1–20)	6 mths: 16 (2–20)			
**Logan 2004 [56]**	*NeADL (Median*, *IQR)*	*NeADL (Median*, *IQR)*	*NeADL* Mean difference (95% CI):		
	Base: 23 (12–31)	Base: 21 (9–35)	*Base*: *NR*		
	10mths: NR	10mths: NR	10mths: 3.94 (-1.52 to 10.30)		
**Parker 2001 [57]**	*Leisure Therapy*	*Control*	*BI*		
	*BI (Median*, *IQR)*	*BI (Median*, *IQR)*	All p = NR		
	Base:18 (15–19)	Base: 18 (16–19)			
	6mths:17 (14–19)	6mths: 17 (15–20)			
	12mths: 17 (14–18)	12mths: 17 (14–20)			
	*NeADL*	*NeADL*	*NeADL*		
	6mths: 33.3 (18.4)	6mths: 33.1 (18.9)	*All p = NR*		
	12mth:32.7 (17.8)	12mth: 33.3 (19.5)			
	*ADL Therapy*				
	*BI (Median*, *IQR)*				
	Base: 18 (16–20)				
	6mths: 18 (15–20)				
	12mths: 17 (14–19)				
	*NeADL*				
	6mths: 34.7 (18.4)				
	12mth:34.1 (19.1)				
**Sackley 2006 [48]**	*BI (Mean*, *SD)*	*BI (Mean*, *SD)*	*BI* Mean difference (95% CI):		
	Base: 10.1 (5.7)	Base: 9.5 (5.2)	Base: NR		
	6mths: 10.21 (5.9)	6mths: 8.09 (4.45)	6mths: 1.5 (-0.5, 3.5) p = .07		
**Walker 1996 [47]**	*Rivermead ADL (Mean*, *SD)*	*Rivermead ADL (Mean*, *SD)*	*Rivermead ADL*		
	Base: 8.4 (3.2)	Base: 7 (4)	*Base*: *NR*		
	6mth: NR	6mth: NR	6mth p = NR		
**Walker 1999 [58]**	*BI (Median*, *IQR)*	*BI (Median*, *IQR)*	*BI Mean difference (95% CI)*:		
	Base: 18 (15–20)	Base: 18(15–20)	*Base*: *NR*		
	6mths: 20 (18–20)	6mths: 18 (16–20)	6mths: 1 (0, 1) p = .002		
	*NeADL (Median*, *IQR)*	*NeADL (Median*, *IQR)*	*NeADL Mean difference (95% CI)*:		
	Base:10 (4–15)	Base: 11 (3–16)	*Base*: *NR*		
	6mths: 16 (11–18.75)	6mths: 12 (6–17)	6mths: 3 (1,4) p = .009		

ADL: Activities of Daily Living Base: Baseline BI: Barthel Index FIM: Functional Independence Measure IQR: Inter-quartile Range Mths: months NeADL: Nottingham Extended Activitities of Daily Living NR: Not reported NS: Not significant Rivermead ADL: Rivermead Activities of Daily Living SD: Standard Deviation

In consideration of several risks of bias, heterogeneity between trials, and mixed findings, the quality of these findings is considered low using the GRADE rating system (see Table 10). This review proposes that increased OT may be beneficial regarding ADL and so should therefore be considered for older stroke survivors.

#### What is the effectiveness of alternative occupational therapy techniques upon older stroke survivors ADL recovery against traditional ADL based occupational therapy?

Regarding comparing alternative OT approaches, two studies presented relevant ADL data (Table 11). Jongbloed *et*. *al*. (1989) found no statistically significant differences between sensorimotor occupational therapy and ADL-based occupational therapy [53]. Donkervoort *et*. *al*. *(*2001) found a statistically significant difference between groups at eight-week follow-up (p<0.01) favouring strategy training over ADL training [51]. However, by five months the different no longer reached significance (p = .11). Very limited evidence and a low GRADE assessment score (see Table 11), means that we are unable to recommend one OT approach above another in relation to older stroke survivors ADL recovery.

**Table 11:** Results of studies comparing alternative occupational therapy approaches upon older stroke survivors ADL recovery.

Study	Intervention	Control	p-value	GRADE Score	GRADE Comment
**Donkervoort 2001 [50]**	*BI (Mean change*, *SD)*	*BI (Mean change*, *SD)*	*BI*: *Mean difference (95% CI)*:	Low	a. The two trial interventions substantially differ in relation to content and techniques used b. Small overall sample size c. One of two studies reported significant results favouring an alternative OT intervention against usual care, although the benefit was no longer significant at follow-up (5months).
	Base: 10.7 (4.9)	Base: 11.2 (5.0)	Base: NR		
	8wks: 2.44 (3.14)	8wks: 1.15 (2.53)	8wks: 1.30 (*0*.*36*, *2*.*24)* p < .01, effect size .47		
	5mths: 3.00 (4.11)	5mths: 2.83 (3.29)	5mths: 0.18 *(1*.*14*,*1*.*49)* p = .11, effect size .05		
**Jongbloed 1989 [52]**	*BI (Mean*, *no SD given)*	*BI (Mean*, *no SD given)*	*BI*		
	Base: 56.05	Base: 51.17	All p = NS		
	4wks: 68.95	4wks: 64.78			
	8wks: 75.57	8wks: 74.71			

BI = Barthel Index Mths = Months NS = Not Significant SD = Standard Deviation Wks = Weeks

#### What is the effectiveness of additional task-specific occupational therapy versus usual occupational therapy upon older stroke survivors disability scores?

Only one RCT, Parker *et*.*al*. (2001), explored the impact of additional OT upon disability scores [57]. They compared usual OT rehabilitation against participants receiving additional task specific training in either leisure activity engagement or self-care activities. Using the Oxford Handicap Scale, measured at six and twelve months post-intervention, the authors found no significant differences between the groups at any assessment (p-values not reported) [57]. Due to unblinded participants, the evidence was downgraded to moderate using the GRADE approach. At present, we are unable to recommend the use of additional OT to improve older stroke survivors post-stroke disability scores

### Optical

#### Studies

Three RCTs present findings in relation to the use of interventions designed to address visual neglect experienced by older stroke survivors. Studies were undertaken in the U.K. (n = 1), Japan (n = 1) and China (n = 1).

#### Participants

In total, 123 participants were randomised, of which 55% (n = 68) were male. Participants mean age ranged from 66 (SD 11.5) [59] to 77.9 (SD 8.6) [60]. Time between stroke and intervention ranged from a median of six days (IQR 2-14days) [60] to 67.1 days (SD 18.4) [59]. Participant characteristics are summarised in Table 12.

**Table 12:** Participant characteristics and study descriptions of included optical interventions.

Study	Arm	No. of Participants	Male/ Female	Age (Yrs, Mean, SD)	Time post-stroke (Mean, SD)	Description	Timing	Treatment Length
**Kalra 1997 [60]**	I	50	20/30	77.9 (8.6)	6 days (2–14)*	A spatio-motor cueing approach based on the attentional-motor integration model.	NR	NR
	C			76.1 (9.9)		Conventional therapy.	NR	NR
**Mizuno 2011 [59]**	I	38	27/11	66 (11.5)	67.1 days (18.4)	Repetitive pointing task using prism glasses.	Two 20-minute sessions per day, 5 days per week.	2 weeks
	C			66.6 (7.7)		Control participants wore non-prism plastic glasses and perform the same pointing tasks as intervention group.	Two 20-minute sessions per day, 5 days per week.	2 weeks
**Tsang 2009 [61]**	I	35	21/14	70.5 (9.3)	22.2 days (15.87)	Conventional occupational therapy programme training in activities of daily living and upper extremity remedial tasks whilst wearing right half-field eye patching glasses.	Five one-hour sessions per week	4 weeks
	C			71.8 (5.26)		Same conventional occupational therapy programme but without the eye patch glasses	Five one-hour sessions per week	4 weeks

*Median and IQR reported by study

C: Control I:Intervention IQR: Interquartile Range NR: Not reported

#### Interventions

Interventions ranged from spatio-motor cueing [60], the wearing of prism glasses [59] or eye patching [61]. Intervention descriptions are presented in Table 12.

#### Risk of bias

Two of the three studies had no or inadequate participant blinding, although all three had adequate assessor blinding. Additionally, each of the three studies methods of allocation concealment were inadequately described.

#### Can optical interventions which target stroke related visual neglect influence stroke survivors ADL recovery in comparison to those receiving conventional rehabilitation only?

All three optical intervention studies measured ADL as an outcome (results presented in Table 13) [59–61]. However, not one of the studies reported significant benefit favouring the optical intervention. Overall, there is no evidence to support the use of interventions targeting visual neglect to improve ADL recovery amongst older stroke survivors. Concerns regarding bias, and the heterogeneity of the trials, results in the quality of evidence being considered very low (see Table 13). According to GRADE, this means that further studies are very likely to change the estimated effect. At present, the use of these approaches cannot be recommended.

**Table 13:** Results of studies investigating the impact of optical interventions upon older stroke survivors ADL recovery.

Study	Intervention	Control	p-value	GRADE Score	GRADE Comment
Kalra 1997 [60]	*BI (Median*, *IQR)*	*BI (Median*, *IQR)*	*BI*	Very Low	a. 2 of the 3 studies did not blind subjects, 2 of the 3 studies did not clarify methods of concealment, and none of the studies could be verified for reporting bias against their protocols. b. Different types of visual interventions were compared delivered each with a different treatment duration and follow up assessment time points. c. Small sample sizes d. None of the three studies demonstrated significant benefit upon ADL from optical interventions against usual care or sham interventions.
	Base: 4 (2–12)	Base: 4 (2–7)	Base: p = NS		
	Discharge: 16 (NR)	Discharge: 14 (NR)	Discharge: p = < .01		
	12wks: 14 (8–18)	12wks: 12.5 (4–16)	12wks: p = NS		
Mizuno 2011 [59]	FIM	FIM	*FIM*		
	NR	NR	P = NS		
Tsang 2009 [61]	*FIM (Mean*, *SD)*	*FIM (Mean*, *SD)*	*FIM*		
	Base: 56.24 (15.72)	Base: 46.94 (16.15)	Base: p = .099		
	Change: 16.0 (14.24)	Change: 12.41 (14.21)	Change: p = .467		

ADL: Activities of Daily Living Base: Baseline BI: Barthel Index FIM: Functional Independence Measure IQR: Interquartile Range NR: Not Reported NS: Not Significant SD: Standard Deviation Wks: Weeks

### Physiotherapy

#### Studies

Seventeen RCTs presented findings in relation to physiotherapy (PT) interventions designed to improve ADL and/or disability recovery of older stroke survivors. Seven were conducted in the UK, three in the USA, two in Norway and one each from Finland, Holland, Ireland, Israel and Korea.

#### Participants

In total, 1354 older stroke survivors participated in these trials, of which approximately 60.7% (n = 823) were male (N.B. Dickstein *et*. *al*., 1996 [62] and Duncan *et*. *al*., 1998 [63] did not present participants sex information). Participant characteristics are presented in Table 14 .

**Table 14:** Participant characteristics and study descriptions of included physiotherapy interventions.

Study	Arm	No. of Participants	Male/ Female	Age (Yrs, Mean, SD)	Time post-stroke (Mean, SD)	Description	Timing	Treatment Length
Askin 2010 [64]	I	62	29/33	75.4 (7.9)	14.4 (7.4) days	Additional motor training involving reaching tasks in sitting and standing positions, sit-to-stand, step tasks, and walking tasks.	30 to 50-minute sessions, 3 times per week for first month and once per week for the next 2 months	12 weeks
	C			77.6 (9.6)		Usual care.	30-minute sessions, twice per day, five days per week.	12 weeks
Dickstein 1986 [62]	I1	131	NR	All: 70.5 (7.65)	NR	Proprioceptive neuromuscular facilitation techniques.	30 to 45-minute sessions, 5 sessions per week	NR
	I2					Bobath techniques.	30 to 45-minute sessions, 5 sessions per week	NR
	C					Conventional treatment.	30 to 45-minute sessions, 5 sessions per week	NR
Duncan 2003 [65] & Studenski 2005 [66]	I	100	50/50	68.5 (9)	77.5 (28.7) days	Home based progressive exercise programme focusing upon strength, balance and endurance, and encouraging use of effected limb.	3 sessions per week	12 weeks
	C			70.4 (11.3)		Usual care plus a visit from research team every second week to provide health education.	1 session every second week	12 weeks
Duncan 1998 [63]	I	20	NR	67.3 (9.6)	66 days (no SD)	Home based exercise program which included assistive and resistive exercises using Proprioceptive Neuromuscular Facilitation Patterns or Theraband exercises to the major muscle groups of the upper and lower extremities.	Three 90-minute sessions per week.	8 weeks
	C			67.8 (7.8)		Usual care and visited by a research assistant every 2 weeks to assess the participants’ exercise and activity level.	Varied	8 weeks
Galvin 2011 [67]	I	40	20/20	69.95 (11.69)	19.7 (3) days	Individualized programmes which comprised of training a family member the skills necessary to carry out the additional exercises. The emphasis of the program was on achieving stability and improving gait velocity and lower limb strength.	35 minutes per day (no. of sessions NR)	NR
	C			63.15 (13.3)		Usual care.	NR	NR
Gelber 1995 [68]	I	27	13 / 14	73.8 (2)	11.3 (1.1) days	Neurodevelopmental training (NDT) which stresses inhibition of abnormal muscle tone and initiation of normal (good quality) motor movements with progression through developmental sequences prior to advancing to functional activities.	NR	NR
	C			69.8 (2.9)		Traditional Functional Retraining (TFR) which stresses practising functional tasks as early as possible even in the presence of spasticity or abnormal postures.	NR	NR
GAPS 2004 [69]	I	70	41 / 29	68 (11)	NR	Additional physiotherapy input (aiming to approximately double the total daily physiotherapy time)	60 to 80-minute sessions, five sessions per week	Mean sessions per participant 43 (95% CI 35–51)
	C			67 (10)		Usual physiotherapy input.	30 to 40-minute sessions, five sessions per week	Mean sessions per participant 32 (95% CI 24–40)
Green 2002 [70]	I	170	95 /75	71.5 (8.7)	NR	All participants were treated with a problem-solving approach at home or in outpatient rehabilitation centres.	3 to 15 sessions over 13 weeks	13 weeks
	C			73.5(8.3)		No intervention.	NR	NR
Kwakkel 1999 [71] & 2002 [6]	I1	101	43/ 58	69.0 (9.8)	7.2 (2.8) days	Additional arm training applied by local physical and occupational therapists plus usual care (15 minutes per day leg rehabilitation, 15 minutes per day arm rehabilitation, and 90 minutes per week ADL training by an occupational therapist).	30 minutes per session, 5 sessions per week (and 4 hours per week usual rehabilitation)	20 weeks
	I2			64.5 (9.7)		Additional leg training applied by local physical and occupational therapists and usual care (15 minutes per day leg rehabilitation, 15 minutes per day arm rehabilitation, and 90 minutes per week ADL training by an occupational therapist).	30 minutes per session, 5 sessions per week (and 4 hours per week usual rehabilitation)	20 weeks
	C			64.1 (15.0)		Immobilisation of the paretic arm and leg by means of an inflatable pressure splint which was applied with the participant in supine position and usual care (15 minutes per day leg rehabilitation, 15 minutes per day arm rehabilitation, and 90 minutes per week ADL training by an occupational therapist).	30 minutes per session, 5 sessions per week (and 4 hours per week usual rehabilitation)	20 weeks
Kim 2016 [72]	I	20	6/4	65.2 (10.1)	30.1 (21.8) days	Group circuit training program conducted under PT supervision. Includes a warm up, five 15-minute exercise sessions, with 1 –minute rest in between each, and ending with a cool down. Exercises include trunk exercises, sitting exercises, sit-stand exercises, walking exercises, aerobic exercise and strength training.	One 90 minute session per day, five days per week	4 weeks
	C		7/3	66.0 (8.8)	29.9 (20.3) days	Individual PT sessions following conventional neuro-developmental treatment approach.	Two 30 minute sessions	4 weeks
Langhammer 2000 [73] & 2003 [74]	I	61	36 / 25	NR	NR	Motor relearning (no further detail given).	NR	NR
	C			NR		Bobath (no further details)	NR	NR
Lincoln 1999 [75]	I1	282	144 / 138	73 (65–81) *	12 (9–17) days*	The qualified-physiotherapist (QPT) group received standard physiotherapy and in addition were treated for 2 hours per week by a senior research physiotherapist. Additional treatment consisted of facilitation, specific neuromuscular techniques, and functional rehabilitation, broadly based on the Bobath approach.	120 minutes per week	5 weeks
	I2			73 (66–80) *		The assistant-physiotherapist (APT) group received standard physiotherapy but in addition were treated for 2 hours per week by a physiotherapy assistant. Treatment consisted of instruction in correct positioning and care of the arm; passive, assisted, and active movements; and practice of functional activities.	120 minutes per week	5 weeks
	C			73 (64–80) *		Usual care.	30 to 45 minute sessions, 5 sessions per week	5 weeks
Morris 2008 [76]	I	106	61/55	67.9 (13.1)	22.6 (5.6) days	Participants practice 4 different tasks (up to 30 practices per task, per session) with both arms, simultaneously.	20 mins per day, 5 days per week	6 weeks
	C			76.8 (9.9)		As per intervention but are performed with only the paretic arm.	20 mins per day, 5 days per week	6 weeks
Sivenius 1985 [77]	I	95	36/59	71.5 (10.5)	NR	Usual physiotherapy delivered in conventional medical wards and then transferred to a specialist rehabilitation unit for more intensive physiotherapy.	NR	NR
	C			70.1 (9.1)		Usual physiotherapy delivered in conventional medical wards followed by discharge to own or care home.	NR	NR
Sunderland 1992 [78]	I	137	60/77	Sev: 65 (32–88) *	Sev: 8 (2–35) days *	Enhanced therapy for arms which is more intensive and utilises behavioural techniques to encourage active participation. Participants were encouraged to practice between sessions, adhere to self-directed exercise programmes, learn new motor skills and discouraged from avoidance of use of effected arm.	NR	NR
				Mild: 67 (46–90)*				
	C			Sev: 68 (50–82) *	Mild: 9 (1–31) days *	Conventional treatment based on Bobath and Johnstone techniques.	NR	NR
				Mild: 70 (35–84) *				
Van Vliet 2005 [79]	I	120	60 / 60	75 (9.1)	NR	Movement Science Based Therapy (no further details).	NR	NR
	C			73.3 (10.4)		Bobath Therapy (no further details).	NR	NR
Wade 1992 [80]	I	94	47/ 47	72.3 (9.7)	53.1 (29.5) months	Delivered by specialist neuro-rehabilitation physiotherapist and conducted in participants homes with carers present. Focused on problem solving in relation to the mobility issues the participant is experiencing, and setting realistic goals to improve mobility.	NR	Mean no. sessions: 4 (2.5). Range 1–11 sessions per participant
	C			72.0 (10.6)		No intervention.	NR	NR

*Median and IQR given

C: Control I: Intervention I1: Intervention arm 1 I2: Intervention arm 2 NR: Not Reported Sev.: Severe SD: Standard Deviation

#### Interventions

Intervention content, delivery and duration varied widely between studies and each intervention is summarised in Table 14 .

#### Risk of bias

Almost all studies (n = 16) were at risk of bias from unblinded or inadequately blinded participants. This said, most studies (n = 15) had adequate outcome assessor blinding. Several studies were at potential risk from biases resulting from randomisation or allocation methods.

#### Does increased physiotherapy influence ADL recovery of older stroke survivors in comparison to those who receive usual rehabilitation care only?

As summarised in Table 15, of the 10 studies addressing this question, only three reported a significant benefit upon ADL favouring additional PT [65, 67, 77]. Each of these three studies identified this benefit only at an intermediate timepoint. Duncan *et*.*al*. *(*2003) found a significant difference in BI scores at three months favouring PT, but not at six months [65]. Similarly, Galvin *et*.*al*. (2011) and Sivenius *et*. *al*., (1985) also reported results favouring increased PT at in the intermediate term (8 weeks and 3 month respectively), but again differences were not maintained in the long term [67,77].

**Table 15:** Results of studies investigating the impact of additional physiotherapy interventions upon older stroke survivors ADL.

Study	Intervention	Control	p-value	GRADE Score	GRADE Comment
**Askim 2010 [64]**	*BI (Mean*, *SD)*	*BI (Mean*, *SD)*	*BI*	Low	a. Nine of the studies did not have participants blinded, and many studies had one or more unclear risks of other sources of bias. b. Interventions varied in content, intensity and duration substantially, for example while some trials focused upon specific limb impairments, others took a whole-body rehabilitation approach. Some trials were very prescribed in their content, while others were more flexible and individualised programmes. c. Additionally, trials were conducted in a range of settings (inpatient rehab., outpatient and in the participants own home). The duration of interventions ranged from 4 weeks to 20 weeks. d. Two of ten studies found significant benefit in relation to ADL scores favouring the PT intervention. Both studies identified these benefits at intermediate time points and benefits were not sustained at longer term follow-up.
	Base: 72.7 (20.0)	Base: 70.8 (16.2)	*Base*: *NR*		
	6mths: 92.5 (9.7)	6mths: 91.4 (16.9)	6mths p = .48		
**Duncan 1998 [63]**	*BI (Mean*, *no SD reported)*	*BI (Mean*, *no SD reported)*	*BI*		
	Base: 82.5	Base: 82.5	Base: NR		
	8wks: 95.5	8wks: 95.6	8wks p = >.02		
**Duncan 2003 [65]**	*BI (Mean*, *SD)*	*BI (Mean*, *SD)*	*BI*		
	Base: 89.2 (11.8)	Base: 85.9 (11.0)	All between group differences p = ns except at 3mths, where SE of difference = 3.35 (p = < .05)		
	3mths: 94.4 (6.7)	3mths: 89.6 (10.4)			
	6mths: 92.6 (9.5)	6mths: 94.3 (7.8)			
	*LIADL (Mean*, *SD)*	*LIADL (Mean*, *SD)*	*LIADL*		
	Base: 21.2 (3.0)	Base: 20.5 (3.9)	All p = NS		
	3mths: 22.8 (3.2)	3mths: 21.8 (3.9)			
	6mths: 23.2 (3.7)	6mths: 22.4 (4.3)			
**Galvin 2011 [67]**	*BI (Mean*, *SD)*	*BI (Mean*, *SD)*	*BI* Change scores:		
	Base: 56.3 (27)	Base: 65.5 (27.9)	*Base*: *NR*		
	8wks: 88.5 (15.6)	8wks: 81.8 (18.7)	*8wks*: *NR*		
	8wks change 32.3 (24)	8wks change 16.3 (14.2)	8wks change p = .04		
	3mths: 92.3 (13.8)	3mths: 83.3 (19)	*3mths*: *NR*		
	3mths change: 3.8 (8.3)	3mths change: 1.5 (11.6)	3mths change p = .36		
	*NeADL*	*NeADL*	*NeADL Change scores*:		
	Base: NR	Base: NR	Base: NR		
	3mths: 41.5 (15.5)	3mths: 32 (20.7)	*3mths*: *NR*		
	3mths change: 7.6 (8.3)	3mths change: 3.6 (7.8)	3mths change p = .02		
**GAPS 2004 [69]**	*BI (Mean*, *SD)*	*BI (Mean*, *SD)*	*BI (Mean difference*, *95% CI)*		
	Base:11.8 (3.3)	Base: 10.3 (3.1)	Base: NR		
	4wks: 14.6 (3.)	4wks 14.1 (3.7)	4wks: NR		
	3mths: 16.6 (2.8)	3mths: 16.1 (3.3)	3mths: 0.7 (-0.9,2.2) p = .39		
	6mths 16.9 (2.7)	6mths 16.2 (4.2)	6mths: 0.7 (-1.1,2.3) p = .45		
	*NeADL (Mean*, *SD)*	*NeADL (Mean*, *SD)*	*NeADL (Mean difference)*		
	Base: NR	Base: NR	Base: NR		
	3mths: 27.6 (12.8)	3mths: 22.2 (11)	3mths: -4.0 (-9.9, 2.0) p = .19		
	6mths:29.1 (11.5)	6mths: 26.2 (13.1)	6mths: -1.5 (-7.7, 4.6) p = .54		
**Green 2002 [70]**	*BI (Median*, *IQR*)	*BI (Median*, *IQR)*	*BI*		
	Base: 18 (16–19)	Base: 18 (16–19)	All p = NS		
	3mths: 18 (16–19)	3mths: 18 (16–19)			
	6mths: 18 (16–19)	6mths: 18 (16–19)			
	9mths: 18 (16–19)	9mths: 18 (16–20)			
	*FAI (Median*, *IQR)*	*FAI (Median*, *IQR)*	*FAI*		
	Base: 10 (4–17)	Base: 13 (7–20)	All p = NS		
	3mths: 9 (4–15)	3mths: 12 (5–17)			
	6mths: 11 (5–18)	6mths: 15 (6–21)			
	9mths: 12 (5–19·5)	9mths: 14 (6–21)			
**Kwakkel 1999 [71]**	*Arm Training*	*Control*	*BI*		
	*BI (Median*, *IQR)*	*BI (Median*, *IQR)*	*Between group difference*		
	Base: 5 (3–7)	Base: 5.5 (3–7)	Base: NR		
	6wks: 10 (5–13)	6wks: 8.5 (7–13)	6wks: p = NS		
	12wks: 14 (10.75–18)	12wks: 11 (8–18)	12wks: p = NS		
	20wks: 17 (14.25–20)	20wks: 16 (10–19)	20wks: p = < .05 (between upper and lower body groups only)		
	26wks: 17 (11.75–20)	26wks: 17 (10.5–19)	26wks: p = NS		
	*Leg Training*				
	*BI (Median*, *IQR)*				
	Base: 6 (3–8)				
	6wks: 13 (8.75–19)				
	12wks: 17 (13–20)				
	20wks: 19 (16–20)				
	26wks: 19 (15–20)				
**Lincoln 1999 [75]**	*Qualified*	Control	BI		
	*BI (Median*, *IQR)*	BI (Median, IQR)	Between grp:		
	Base: 6 (3–9)	Base: 7 (3–9)	Base: NR		
	5wks: 12 (8–16)	5wks: 13 (7–17)	5wks: p = .66		
	3mths: 14 (7–17)	3mths: 14 (10–19)	3mths: p = .51		
	6mths: 16 (9–18)	6mths: 16 (12–19)	6mths: p = .65		
	*EADL*	*EADL*	*EADL*		
	Base: NR	Base: NR	Base: NR		
	5wks: 5 (2–13)	5wks: 7.5 (3–26)	5wks: p = .31		
	6mths: 15 (5–2)	6mths: 13 (7–33)	6mths: p = .65		
	*Assistant*				
	*BI (Median*, *IQR)*				
	Base: 6 (4–8)				
	5wks: 12 (7–17)				
	3mths: 14 (10–17)				
	6mts: 16 (12–18)				
	*EADL*				
	Base: NR				
	5wks: 6 (4–14)				
	6mths: 14 (6–33)				
**Sivenius 1985 [77]**	*ADL (Mean*, *SE)*	*ADL (Mean*, *SE)*	*ADL*		
	Base: NR	Base: NR	*Relative difference (%)*		
	1wk: 10.5 (1.3)	1wk: 13.6 (1.7)	1wk: p < .05		
	3mths: 21.0 (1.3)	3mths: 16.3 (1.7)	3 months: p < .01		
	6mths: 21.6 (1.2)	6mths: 18.6 (1.5)	*6mths*: *NR*		
	12mths: 21.1 (1.3)	12mths: 18.4 (1.6)	*12mths*: *NR*		
**Wade 1992 [80]**	*BI (Mean*, *SD)*	*BI (Mean*, *SD)*	*BI*		
	Base: 16.3 (3.0)	Base: 17.0 (2.8)	All p = NS		
	3mths: 16.2 (3.1)	3mths:16.7 (3.2)			
	*NeADL (Mean*, *SD)*	*NeADL (Mean*, *SD*)	*NeADL*		
	Base: 25.7 (14.3)	Base: 28.4 (24.4)	All p = NS		
	3mths: 25.5 (13.7)	3mths: 27.4 (15.2)			

ADL: Activities of Daily Living Base: Baseline BI: Barthel Index EADL: Extended Activities of Daily Living FAI: Frenchay Activities Index INT1: Intervention arm 1 INT 2: Intervention arm 2 IQR: Inter-quartile Range LIADL: Lawtons Instrumental Activities of Daily Living Mths: Months NeADL: Nottingham Extended Activities of Daily Living NR: Not Reported NS: Not Significant SD: Standard Deviation SE: Standard Error Wks: Weeks

The degree of heterogeneity between interventions, combined with a number of sources of bias across included studies, resulted in the quality of evidence for this intervention being downgraded to low (see Table 15). According to GRADE further studies are very likely to change the effect estimate. In view of the limited evidence, this review proposes that increased PT may be beneficial regarding ADL recovery in the short term and so should be considered for older stroke survivors.

#### What is the effectiveness of specific physiotherapy approaches versus alternative physiotherapy approaches or usual care upon older stroke survivors ADL recovery?

Nine studies provided results to address this question, summarised in Table 16. Only one study [71] reported any benefit favouring an alternative PT approach, and the benefit was limited. In their three-arm trial, Kwakkel *et*. *al*. (1999) reported a statistically significant difference between additional arm training and leg training, favouring leg training, at the 20 week follow-up (p<0.05). However, no statistically significant differences between the three groups at 26-week or one-year follow-up were found [6].

**Table 16:** Results of studies investigating the impact of alternative physiotherapy interventions upon older stroke survivors ADL.

Study	Intervention	Control	p-value	GRADE Score	GRADE Comment
**Gelber 1995 [68]**	*FIM (Mean*, *SD)*	*FIM (Mean*, *SD)*	*FIM*	Low	a. All studies had unblinded participants and uncertainties regarding other bias types are apparent across other studies. b. All trials compared different types of PT and therefore their content and delivery varied widely. c. Only one of eight studies reported significant results favouring an alternative PT approach against usual care, and this study found this benefit only at an intermediate timepoint, and not immediately after intervention or in longer term follow-up.
	Admission: 77.9 (3.8)	Admission: 82.1 (5.8)	All p = NS		
	Discharge: 101.2 (3.8)	Discharge: 105.3 (4.8)			
	6mths: 106.9 (5.4)	6mths: 117.5 (3.5)			
	12mths:109.6 (4.2)	12mths: 114.8 (5.5)			
	Change: 31.9 (4.0)	Change: 28.9 (4.7)			
**Langhammer 2000 [73]**	*BI (Mean*, *SD)*	*BI (Mean*, *SD)*	*BI*		
	Base: 56 (28)	Base: 46 (36)	Base: p = .32		
	3mths: 83 (25)	3mths: 72 (34)	3mths: p = .20		
	12mths: 68 (41)	12mths: 57 (43)	12mths: p = NS		
	48mths: 45 (44)	48mths: 42 (44)	48mths: p = NS		
**Lincoln 1999 [75]**	*Qualified*	*Control*	*BI*		
	*BI (Median*, *IQR)*	*BI (Median*, *IQR)*			
	5wks: 12 (8–16)	5wks: 13 (7–17)	5wks: .66		
	3mths: 14 (7–17)	3mths 14 (10–19)	3mths: .51		
	6mths: 16 (9–18)	6mths: 16 (12–19)	6mths: .65		
	*eADL*	*eADL*	*eADL*		
	5wks: 5 (2–13)	5wks: 7.5 (3–26)	5wks: p = .31		
	6 mths: **15** (5–28)	6mths: 13 (7–33)	6mths: p = .65		
	*Assistant*				
	*BI (Median*, *IQR)*				
	5wks: 12 (7–17)				
	3mths:14 (10–17)				
	6mths: 16 (12–18)				
	*eADL*				
	5wks: 6 (4–14)				
	6mths: 14 (6–33)				
**Kim 2016 [72]**	*Korean modified BI (Mean*, *SD)*	*Korean modified BI (Mean*, *SD)*	*Korean modified BI*		
	Base: 65.7 (23.3)	Base: 57.4 (22.4)	Base: NS		
	4wks: 87.0 (10.5)	4wks: 85.3 (13.7)	4wks: < .01		
	Change: 21.30 (15.13)	Change: 27.90 (14.93)	Change: NS		
**Kwakkel 1999 [71]**	*Arm Training*	*Control*			
	*BI (Median*, *IQR)*	*BI (Median*, *IQR)*	*BI*		
	6wks: 10 (5–13)	6wks: 8.5 (7–13)	6wks: p = NS		
	12wks: 14 (10.75–18)	12wks: 11 (8–18)	12wks: p = NS		
	20wks: 17 (14.25–20)	20wks: 16 (10–19)	20wks: p = < .05 (between upper and lower body groups only)		
	26wks: 17 (11.75–20)	26wks: 17 (10.5–19)	26wks: p = NS		
	52wks: 15 (12.5.20)	52wks: 17 (14–20)	52wks: p = NS		
	*Leg Training*				
	*BI (Median*, *IQR*				
	6wks: 13 (8.75–19)				
	12wks: 17 (13–20)				
	20wks: 19 (16–20)				
	26wks: 19 (15–20)				
	52wks: 18 (14.5–20)				
**Dickstein 1986 [62]**	*Proprioceptive*	*Control*	*BI*		
	*BI*	*BI*	p = NS		
	NR	NR			
	*Bobath*	*Control*			
	*BI*	*BI*			
	NR	NR			
**Van Vliet 2005 [79]**	*BI (Median*, *IQR)*	*BI (Median*, *IQR)*	*BI*		
	Base: 8 (5–11)	Base: 8 (6–13)	Base: NR		
	1mth: 14 (10–18)	1mth: 15 (12–18)	1mth: p = .40		
	3mths: 17 (14–19)	3mths: 17 (13–19)	3mths: p = .94		
	6mths: 17 (15–19)	6mths: 18 (16–20)	6mths: p = .20		
**Morris 2008 [76]**	*BI (Mean*, *SD)*	*BI (Mean*, *SD)*	*BI*		
	Base: 58.5 (28.5)	Base: 65.7 (23.5)	Base: p = NR		
	6wks: 83 (16.2)	6wks: 85.1 (19.2)	6wks: p = .27		
	18wks: 86 (16.9)	18wks: 86.3 (18.4)	18wks: p = .13		
**Sunderland 1992 [78]**	*BI (Median*, *IQR)*	*BI (Median*, *IQR)*	*BI*		
	Mild Impairment	Mild Impairment	All p = NS		
	Base: 13 (2–20)	Base: 12 (6–20)			
	6mths: 20 (7–20)	6mths: 19 (13–20)			
	Severe Impairment	Severe Impairment			
	Base: 7 (2–20)	Base: 7 (2–19)			
	6mths: 17 (2–20)	6mths: 16 (7–20)			

Base: Baseline BI: Barthel Index eADL: Extended Activities of Daily Living FIM: Functional Independence Measure IQR: Inter-quartile Range NR: Not Reported NS: Not Significant Mths: Months SD: Standard Deviation Wks: Weeks

There is little evidence to suggest that one PT technique benefits stroke survivors ADL recovery more than an alternative technique. GRADE assessment of quality suggests the overall evidence base as being low (see Table 16), largely as a result of substantial heterogeneity between intervention content. Therefore, it is not possible for this review to make any recommendations regarding specific PT approaches to enhance older stroke survivors ADL recovery.

### Psychological therapies

#### Studies

Six RCTs explored the use of psychological therapies amongst older stroke survivors, with three conducted in the UK, one in Holland, one in Australia and one in the USA.

#### Participants

In total, 946 participants were randomised, of which 54.5% (n = 516) were male, with mean ages ranging from 65 (SD 15.1 SD) years [81] to 77.9 (SD 7.4) years [82]. Table 17 presents a summary of participant characteristics.

**Table 17:** Participant characteristics and study descriptions of included psychological therapy interventions.

Study	Arm	No. of Participants	Male/ Female	Age (Yrs, Mean, SD)	Time post-stroke (Mean, SD)	Description	Timing	Treatment Length
**Bradley 1998 [83]**	I	23	12 / 11	Mild: 66.6 (No SD)#	35.6 days *(No SD)*	Physiotherapy with electromyography biofeedback, tailored to the individual’s needs. Electrodes were placed on appropriate muscle groups and participants performed various activities, using the sound and light indicators of the feedback machine to control muscle contraction and relaxation.	3 sessions per week	Max. 18 sessions
				Sev: 72.4 *(No SD)#*				
	C			Mild: 77 (No SD)#		As per intervention, but biofeedback machine was turned away from the participant and therapist.	3 sessions per week	Max. of 18 sessions
				Sev: 68 *(No SD)#*				
**Braun 2012 [82]**	I	36	14 / 22	77.7 (7.2)	6.1 weeks (2.7)	Therapists explained the concept of mental practice and helped participants develop and use imagery techniques focused towards improving motor skills required to drink from a cup, and/ or walk 10m.	NR	6 weeks
	C			77.9 (7.4)		The same rehabilitation programme, but without mental imagery.	NR	6 weeks
**Clark 2003 [84]**	I	62	38 / 24	73.3 (8.5)	39.6 days (18.4)	Counselling and information sessions conducted by a social worker.	Three one-hour sessions delivered at 2 weeks, 2 month and 5 months.	5 months
	C			71.2 (8.8)		No intervention/ Usual care.	NR	NR
**Ertel 2007 [85) & Glass 2004 [87]**	I	291	149 / 142	69.3 (11.1)	NR	Cognitive behaviour therapy (CBT) delivered by a trained psychologist or social worker who met with participant, caregiver, family, friends, and professional caregivers. Sessions aimed to increase self-efficacy, optimise social support, reduce stress and increase problem solving via CBT techniques.	One 60-minute session per week	Up to 16 sessions over 6 months
	C			70.2 (10.9)		Usual care.	NR	NR
**Lincoln 2003 [81]**	I1	123	63 / 60	67.1 (12.7)	< 1 month n = 61 1–3 months n = 27 3–6 months n = 35	Cognitive behavioural therapy sessions delivered by psychiatric nurse which included education, graded task assignment, and identifying unhelpful thoughts/ behaviours.	Ten one-hour sessions	3 months
	I2			66.1 (13.2)		Placebo sessions delivered by psychiatric nurse with no therapeutic interventions.	Ten one-hour sessions	3 months
	C			65 (15.1)		No intervention.	NR	NR
**Watkins 2011 [86]**	I	411	240 / 171	70 (61–78) *	18.5 days * (12–29)	Motivational interviewing sessions where participant concerns, goals, barriers to goals, plans and solutions, were explored.	Thirty to sixty-minute sessions, once per week	4 weeks
	C			70 (61–77) *		No intervention.	NR	NR

*Median and IQR given #Participants were categorised as either mild or severe in terms of impairment.

C: Control I: Intervention I1: Intervention arm 1 I2: Intervention arm 2 NR: Not Reported Sev. Severe SD: Standard Deviation

#### Interventions

The six studies varied in their content and underlying theoretical basis, and included biofeedback [83], mental imagery [82], counselling [84], (cognitive behaviour therapy (CBT) [81, 85] and motivational interviewing [86]. A summary description of each intervention is presented in Table 17.

#### Risk of bias

All studies had a risk of bias arising from either unblinded participants. However, all reported adequate assessor blinding, and all but one had a low risk of bias arising from randomisation or allocation methods.

#### Can psychological therapies influence older stroke survivors ADL recovery in comparison to those who receive usual rehabilitation care or sham treatment only?

Only one of the six studies addressing this question reported a significant improvement in ADL (see Table 18). In the study by Clark *et*. *al*. (2003), intervention participants who received counselling sessions from a trained social worker had a significantly greater improvement in ADL score than control participants at six month follow up [84]. It should, however, be noted that although statistical significance was reached, the difference between groups is arguably small and clinical significance questionable.

**Table 18:** Results of studies investigating the impact of psychological therapy interventions upon older stroke survivors ADL recovery.

Study	Intervention	Control	p-value	GRADE Score	GRADE Comment
Bradley 1998 [83]	*NeADL*	*NeADL*	*NeADL*	Low	a. All trials involved unblinded patients and several other bias risks such as inadequate information regarding randomisation and allocation process, and unexplained missing data, were apparent. b. Type of intervention varies significantly (MI, CBT, Counselling, Biofeedback). c. One of the six studies measured ADL using a different measure from the others, and only one study of six reported a significant result in favour of psychological therapies.
	NR	NR	p = NS		
Braun 2012 [82]	*BI (Mean*, *SD)*	BI (Mean, SD)	*BI (ITT*, *Mean difference*, *95%CI)*		
	Base: 11.17 (4.1)	Base: 12.22 (5.4)	Base: NR		
	6wks: 15 (14.5)	6wks: 14.94 (5.5)	6wks: 0.9 (1.51–3.31) p = 0.46		
	6mths: 15.55 (4.2)	6mths: 15.56 (5.3)	6mths: 0.34 (-2.69, 3.37) p = 0.83		
Clark 2003 [84]	*BI (Mean*, *SD)*	*BI (Mean*, *SD)*	*BI*		
	Base: 16.4 (3.7)	Base: 16.6 (2.6)	Base: NR		
	6mths: 18.7 (2.0)	6mths: 17.4 (3.9)	6mths: p = < .05		
Ertel 2007 [85] & Glass 2004 [87]	*BI (Mean*, *SD)*	*BI (Mean*, *SD)*	*BI*		
	Base: 65.5 (20.4)	Base: 65.6 (19.3)	Base: p = : .96		
	3mths: 87.1 (14.2)	3mths: 85.4 (17.2)	3mths: p = NS		
	6mths: 89.5 (14.1)	6mths: 86.5 (18.2)	6mths: p = NS		
Lincoln 2003 [81]	*CBT eADL (Median*, *IQR)*	*Control eADL (Median*, *IQR)*	*eADL*		
	Base: 21 (14–34)	Base: 27 (15–38)	Base: p = .40		
	3mths: 29 (21–39)	3mths: 35 (20–45)	3mths: p = .70		
	6mths: 30 (17–43)	6mths: 30 (21–43)	6mths: p = .90		
	*Placebo eADL (Median*, *IQR)*				
	Base: 26.5 (18–35)				
	3mths: 29 (18–44)				
	6mths: 31.5 (22–44)				
Watkins 2011 [86]	*BI (no*. *of persons categorised as mild*, *moderate or severe*)	*BI (no*. *of persons categorised as mild*, *moderate or severe)*	*BI* All p = NS		
	Base:	Base:			
	Mild: 100 (49%)	Mild: 99 (47.8%)			
	Moderate: 61 (30%)	Moderate: 62 (30%)			
	Poor: 43 (21%)	Poor: 46 (22%)			
	3mths:	3mths:			
	Mild: 105 (59.5%)	Mild: 105 (58.7%)			
	Moderate: 54 (30.7%)	Moderate: 51 (28.5%)			
	Poor: 13 (7.4%)	Poor: 11 (6.1%)			
	Dead: 4 (2.3%)	Dead: 12 (6.7%)			

Base: Baseline BI: Barthel Index CI: Confidence Interval CBT: Cognitive Behavioural Therapy eADL: Extended Activities of Daily Living IQR: Interquartile Range ITT: Intension to Treat MThs: Months NeADL: Nottingham extended Activities of Daily Living, NR: Not Reported NS: Not Significant SD: Standard Deviation Wks: Weeks

At present, the evidence was assessed by GRADE to be of low quality and does not support a recommendation for the use of psychological therapies to improve ADL recovery in older stroke survivors.

#### Can psychological therapies affect post-stroke disability in older stroke survivors in comparison to those who receive usual rehabilitation care or sham treatment only?

Only one study, Lincoln *et*. *al*. *(*2003), explored the use of CBT upon post stroke disability amongst older stroke survivors [81]. The three arm trial compared CBT, a sham talking treatment and usual care [81]. Using the London Handicap Scale (LHS), the study identified no significant difference in LHS scores between the groups at baseline, 3 or 6 months. The evidence was assessed by GRADE to be of low quality and therefore at this time, there is no evidence to recommend the use of CBT in older stroke survivor

### Self-management education

#### Studies

Six RCTs presented findings in relation to self-management education interventions targeting either older stroke survivors ADL recovery or disability. Four were conducted in the UK and one each from Sweden and Israel.

#### Participants

In total, 1012 older stroke survivors participated in these trials, of which 531 (52.5%) were male. Participant characteristics are summarised in Table 19.

**Table 19:** Participant characteristics and study descriptions of included self-management education interventions.

Study	Arm	No. of Participants	Male/ Female	Age (Yrs, Mean, SD)	Time post-stroke (Mean, SD)	Description	Timing	Treatment Length
**Forster & Young 1996 [88]**	I	240	127/ 113	73 (60–94) *	NR	Programme of home visits conducted by specialist nurses. Participants were provided stroke information and encouraged to identify problems and solutions, to set goals, and return to social activities.	Minimum 6 visits in first 6 months.	Up to 12 months.
	C			73 (60–90) *		Usual care.	NR	NR
**Guidetti & Ytterberg, 2010 [89] & 2011 [90]**	I	40	17/ 23	66 (14)	NR	A 9-step programme involving the development of an individualised self-care plan tailored to participants needs, goal setting, problem solving, and practice of desired activities.	NR	NR
	C			69 (15)		Usual self-care training as covered in standard rehabilitation care within the unit.	NR	NR
**Johnston 2007 [91]**	I	203	124/ 79	68.96 (12.64)	NR	Participants (stroke survivor and their carer) were provided a workbook to complete over 5 weeks with support from a researcher. Workbook included general stroke and recovery information, coping skills, self-management, diary sheets, and relaxation exercises.	5 contacts	5 weeks
	C			68.79 (12.02)		Usual care.	NR	NR
**Nir 2004 [92]**	I	155	80/ 75	72.3 (6.8)	NR	Nursing self-care intervention started whilst participant was in the rehabilitation unit, and then continued at home. Intervention involved building confidence in the nurse facilitator, challenging participant attitudes/ beliefs/ knowledge of stroke, increasing self-care skills and increasing participant responsibility for health and rehab.	One to two-hour session weekly	12 weeks
	C			73.8 (7.6)		Usual care.	NR	NR
**Rodgers 1999 [93]**	I	204	97/ 107	74 (36–94) *	NR	Begins with a small group education session for inpatients and their carers and is followed by six sessions at home. Programme aims to improve knowledge of stroke, treatments, and services, provide advice and an opportunity to ask questions and gain support. Led by one member of multi-disciplinary team with input from nursing, PT, OT, speech/language therapy, psychology, social work, carers, and a stroke club.	One hour session per week	7 weeks
	C			76 (36–95)*		Usual care.	NR	NR
**Smith, Forster & Young 2004 [94]**	I	170	86/ 84	75 (31–91) *	NR	Participants were given a stroke recovery manual covering causation, consequences, recovery, financial benefits, services, and carer information. Participants also attended meetings every two weeks with the multidisciplinary team to discuss progress.	One to five 20-minute sessions	NR
	C			74 (50–92) *		Usual care.	NR	NR

*Median and IQR given

C: Control I: Intervention I1: Intervention arm 1 I2: Intervention arm 2 NR: Not Reported OT: Occupational Therapy PT: Physiotherapy SD: Standard Deviation

#### Interventions

Each intervention focused upon providing education and developing self-management skills and plans, but their content and mode of delivery varied, as described in Table 19.

#### Risk of bias

Each RCT had at least one significant risk of bias, most commonly from unblinded or inadequately blinded participants. Several studies also were at high risk of bias arising from their randomisation and allocation methods.

#### Do self-management education interventions influence ADL recovery of older stroke survivors in comparison to those who receive usual rehabilitation care only?

Six studies explored if a self-management education intervention could affect post stroke ADL recovery. Results are summarised in Table 20. Only one of the six studies, Nir *et*. *al*. 2004, identified a significant improvement in FIM scores following the intervention against a control group receiving usual care [92]. However, this study also measured ADL using the IADL questionnaire and found no significant difference between groups at any follow-up.

**Table 20:** Results of studies investigating the impact of self-management education interventions upon older stroke survivors ADL.

Study	Intervention	Control	p-value	GRADE Score	GRADE Comment
**Nir 2004 [92]**	*FIM (Mean*, *no SD given)*	*FIM (Mean*, *no SD given)*	*FIM*	Low	a. All studies involved unblinded participants and some aspects such as randomisation and allocation in some studies were unclear. b. Substantial variation in delivery, content and duration of interventions c. A variety of measures were used between the studies, and only one of six studies reported significant results in favour of the self-management intervention.
	Base: 77	Base: 75	*Base*: *NR*		
	3mths: 103	3mths: 90	*3mths*: *NR*		
	6mths: 104	6mths: 93	6mths: p = < .001		
	*IADL (Mean*, *no SD given)*	*IADL (Mean*, *no SD given)*	*IADL*		
	Base: 16	Base: 17	*Base*: *NR*		
	3mths: 25	3mths: 28	*3mths*: *NR*		
	6mths: 24	6mths: 27	6mths: p = 0.45		
**Johnston 2007 [91]**	*BI (Mean*, *SD)*	*BI (Mean*, *SD)*	*BI*		
	Base: 1.57 (0.73)	Base: 1.5 (0.63)	All p = NS		
	5wks: 1.44 (0.65)	5wks: 1.43 (0.59)			
	6mths: 1.43 (0.68)	6mths: 1.39 (0.61)			
**Smith 2004 [94]**	*BI (Median*, *IQR)*	*BI (Median*, *IQR)*	*BI*		
	Base:6 (0–27)	Base: 6 (0–17)	All p = NS		
	3mths:14 (0–20)	3mths: 13 (1–20)			
	6mths:15 (0–20)	6mths: 15 (0–20)			
	*FAI (Median*, *IQR)*	*FAI (Median*, *IQR)*	*FAI*		
	Base: NR	Base: NR	All p = NS		
	3mths: 1 (0–30)	3mths: 0 (0–23)			
	6mths: 5 (0–32)	6mths: 3 (0–33)			
**Rodgers 1999 [93]**	*NeADL*	*NeADL*	*NeADL*		
	No data presented	No data presented	No data presented but all p = NS		
**Forster 1996 [88]**	*BI (Median*, *IQR)*	*BI (Median*, *IQR)*	*BI*		
	Base: 17 (12–19)	Base: 16 (12–18)	All p = NS		
	3mths: 17 (14–19)	3mths: 17 (14–19)			
	6mths: 18 (13–20)	6mths: 17 (13–19)			
	12mths: 18 (14–19)	12mths: 17 (13–19)			
	*FAI (Median*, *IQR)*	*FAI (Median*, *IQR)*	*FAI*		
	Base:27 (20–31)	Base: 26 (20–31)	All p = NS		
	3mths: 8 (3–13)	3mths: 6 (3–12)			
	6mths: 13 (5–20)	6mths: 10 (4–17)			
	12mths: 12 (6–19)	12mths: 10 (5–20)			
**Guidetti 2010 [89] & 2011 [90]**	*BI (Median*, *IQR)*	*BI (Median*, *IQR)*	*BI*		
	Base: 55 (35–70)	Base: 25 (15–50)	p = NR		
	Unweighted	Unweighted	Unweighted		
	3mths: 93 (85–100)	3mths: 90 (50–100)	3mths p = .32		
	Weighted	Weighted	Weighted		
	3mths: 41 (32–45)	3mths: 37 (14–42)	3mths p = .34		
	6mths: 41 (34–45)	6mths: 38 (13–42)	6mths: NR		
	12mths: 41 (32–45)	12mths: 34 (9–45)	12mths: NR		
	*FIM A-M (Median*, *IQR)*	*FIM A-M (Median*, *IQR)*	*FIM A-M*		
	Base: 64 (44.5–74)	Base: 48 (31–63)	Base: NR		
	3mths: 83 (81–88.5)	3mths: 79 (62–86.5)	3mths: NR		
	12mths: 84 (79–91)	12mths: 82 (70–87)	12mths: p = .25		

Base: Baseline BI: Barthel Index FAI: Frenchay Activities Index FIM: Functional Independence Measure FIM A-M: Shortened version of the Functional Independence Measure IADL: Instrumental Activities of Daily Living NeADL: Nottingham extended Activities of Daily Living NS: Not Significant Mths: Months Wks: Wee

In summary, while several studies have explored the impact of self-management education interventions upon post-stroke ADL recovery, there is very little evidence to support their use. The evidence was considered to be of low quality and therefore we cannot recommend these interventions to benefit older stroke survivors.

#### Do self-management education interventions influence post-stroke disability scores of older stroke survivors in comparison to those who receive usual rehabilitation care?

Two studies explored the impact of self-management education interventions upon post-stroke disability score. One study, Rodgers et al 1999, did not report any original data but we are informed that there were no significant differences between the disability scores of the intervention and control participants [93]. In the study by Smith et al (2004), both intervention and control groups were found to improve their level of disability post-stroke, as measured by the London Handicap Score, but the difference between those who participated in the self-management education intervention, and those who did not, was not significant [94]. There is presently no evidence to support the use of self-management education programmes for older stroke survivors to improve post-stroke disability. An assessment of the quality of these studies, using the GRADE approach, rates the overall quality as very low. Principally, this is due to small sample size, heterogeneity between the RCTs and risk of bias.

### Videogames

#### Studies

One RCT investigating the role of videogames in the treatment of older stroke survivors was identified. This study, reported by Lee *et*. *al*. (2013) [95], was conducted in Korea, but is considered at high risk of bias due to a lack of reporting study methodological information.

#### Participants

The trial randomised 14 participants, 9 (64%) of whom were male. The mean age of the intervention group was 71.71 (SD 9.14) years, and the control group 76.43 (SD 5.8) years. The intervention group had a mean time between stroke onset and commencement of intervention of 7.29 months (SD 1.38), in comparison to the control group mean of 8.29 months (SD 3.4).

#### Intervention

The 6-week video gaming intervention involved participants being asked to choose two games on an Xbox Kinect games console to play while sitting or standing. The games were played in a separate room without distractions. The games were designed to complement conventional OT therapy and participants asked to participate in these 60-minute sessions three times per week. Control participants received usual rehabilitation care only.

#### Risk of bias

For almost all bias types, this study was rated as being unclear due to insufficient reporting.

#### Can the use of video games in addition to conventional occupational therapy delivered within inpatient rehabilitation care influence stroke survivors ADL recovery against usual occupational therapy alone?

The small study (n = 14) by Lee *et*. *al*. (2013) found that both intervention and control groups significantly improved their FIM scores at the post-intervention assessment (Intervention group post-test: 71.42, SD 15 v control group post-test: 61.24, SD 11.9), but no significant difference between the groups in relation to their degree of improvement or final scores were identified (p values not given) [95]. Based upon one small study (n = 14), with a high risk of bias, which demonstrated no benefit, means that we are unable to recommend the use of videogames to improve ADL recovery amongst older stroke survivors. Using the GRADE system the results suggest the evidence is of low quality, meaning that further studies are very likely to change the effect estimate.

### Wheelchair

#### Studies

Only one RCT, conducted by Barrett *et*. *al*. 2001, was identified which investigated if self-propulsion of a wheelchair improved ADL recovery against non-self-propulsion [96]. It was conducted across two inpatient rehabilitation units within one UK hospital had a high risk of bias.

#### Participants

This study involved forty participants, 24 (60%) of whom were male. The mean age of the intervention group was 67.5 years (SD 10.4) versus the control group of 66.7 years (SD 12.0). Intervention participants had a mean time between stroke and intervention of 16.1 days (SD 8.8) versus control participants 15.6 days (SD 8.1).

#### Intervention

Intervention participants were encouraged to self-propel a wheelchair following instruction by a physiotherapist on how to self-propel. Intervention participants were encouraged by ward staff members to self-propel as much as they could, with weekly reminders from the study team. This encouragement continued until discharge or for a maximum of 8 weeks. Control participants were also provided a wheelchair but were actively discouraged from self-propulsion.

#### Risk of bias

Lack of blinding and insufficient reporting resulted in this study being considered to be of unclear bias risk.

#### Can encouragement to self-propel a wheelchair influence ADL outcome amongst inpatient stroke survivors against discouragement to self-propel a wheelchair?

Only one paper, Barrett *et*. *al*. (2001), addresses this question [96]. Both groups improved their BI scores at 3 months (11.4 (4.0 SD) v 9.8 (5.0 SD)) and 12 months (11.9 (5.2 SD) v 11.9 (4.1 SD) but no significant difference between the groups was identified (no p value reported). Similarly, both groups improved their NeADL scores at 3 months (5.8 (5.2 SD) v 5.3 (4.0 SD)) and at 12 months (7.1 (4.7 SD) v 8.0 (5.3 SD) but again the difference between groups was not significant. Therefore, there is no evidence at present to recommend the encouragement of wheelchair propulsion as part of stroke rehabilitation care amongst older stroke survivors. With the bias risks imposed (no participant blinding, concerns about selection bias and small sample size) the evidence has been rated as low using the GRADE system meaning that further studies are very likely to have an important impact on the estimate of benefit.

### Summary of recommendations

Table 21 presents a summary of the recommendations this study proposes based on the identified evidence.

**Table 21:** Summary of recommendations for non-pharmacological interventions for older stroke survivors rehabilitation.

Category	Recommendation
**Acupuncture**	There is very limited evidence to show that acupuncture can benefit older stroke survivors ADL and no evidence to support its benefit upon Disability. A low GRADE quality assessment score, combined with this limited evidence, means that we cannot recommend acupuncture for older stroke survivors.
**Caregiver Training**	There was very limited evidence to show that caregiver training can benefit older stroke survivors ADL, and no evidence to support any benefit upon disability scores. Only one study was considered in this category and was given a GRADE quality assessment score of low. Therefore, we are unable to recommend caregiver training to benefit older stroke survivors.
**CIMT**	There was no evidence to show that CIMT improves ADL performance and the study was assessed by GRADE to be of low quality. Therefore, we cannot recommend the use of CIMT for older stroke survivors.
**Device assisted PT**	There is very limited evidence to support the use of device assisted PT to enhance older stroke survivors ADL. The quality of the evidence was assessed by GRADE and found to be low. Therefore, we cannot recommend device assisted PT for older stroke survivors
**Music Therapy**	Evidence from one study does not allow us to support the use of music therapy to enhance older stroke survivors ADL. The quality of the evidence was assessed by GRADE and found to be low and therefore we cannot recommend the use of music therapy to benefit older stroke survivors.
**Nerve Stimulation**	There was very limited evidence to show that nerve stimulation can benefit older stroke survivors ADL. The quality assessment score was very low. Therefore, we cannot recommend nerve stimulation to benefit older stroke survivors ADL.
**OT**	There is evidence to show that additional OT can benefit older stroke survivors ADL but no evidence to show that alternative OT approaches can benefit older stroke survivors ADL. GRADE quality assessment suggests the evidence for to be of a low score. Therefore, the use of additional OT can be recommended as it may benefit older stroke survivors ADL. However, alternative OT approaches cannot be recommended for older stroke survivors ADL. In relation to disability, there was no evidence to suggest that additional occupational therapy can improve post-stroke disability. The evidence for disability was given a GRADE quality assessment score of moderate. Therefore, we are unable to recommend additional occupational therapy to benefit older stroke survivors’ disability.
**Optical**	There was no evidence to show that optical interventions can benefit older stroke survivors ADL and quality assessment of included studies was awarded a GRADE score of very low. Therefore, we are unable to recommend optical therapies to benefit older stroke survivors.
**Psychological Therapies**	There is very limited evidence to show that psychological therapies can benefit older stroke survivors ADL and no evidence to support its benefit upon disability. GRADE quality assessment was scored as low. Therefore, we cannot recommend psychological therapies to benefit older stroke survivors.
**PT**	There is some evidence to show that additional PT can benefit older stroke survivors ADL but no evidence to show that alternative PT approaches can benefit older stroke survivors ADL. GRADE quality assessment suggests the evidence for ADL to be of a low score. Therefore, the use of additional PT can be recommended as it may benefit older stroke survivors ADL. However alternative approaches to PT cannot be recommended for older stroke survivors.
**Self-management Education**	There is very limited evidence to show that self-management education programmes can benefit older stroke survivors ADL and no evidence to support any benefit upon disability. GRADE quality assessment suggests the evidence for ADL to be of low quality, and for disability, very-low, quality. Therefore we cannot recommend self-management education programmes to benefit older stroke survivors.
**Videogames**	There was no evidence to show that videogames can benefit older stroke survivors ADL and quality assessment of the one study in this category was given a GRADE score of very low. Therefore, we are unable to recommend videogames to benefit older stroke survivors.
**Wheelchair Use**	There was no evidence to show that wheelchair self-propulsion can benefit older stroke survivors ADL and the quality assessment of the 1 study in this category was scored as very low. Therefore, we are unable to recommend wheelchair self-propulsion to benefit older stroke survivors.

ADL: Activities of Daily Living CIMT: Constraint Induced Movement Therapy GRADE: Grading of Recommendations Assessment, Development and Evaluation OT: Occupational Therapy PT: Physiotherapy

## Discussion

### Acupuncture

Acupuncture is rarely mentioned as a therapy for stroke survivors within the guidelines. The Royal College of Physicians [4] refer to the limited evidence for acupuncture in the treatment of post-stroke dysphagia. The Scottish Intercollegiate Guidelines Network [5] state that they do not recommend acupuncture for the treatment of post-stroke pain syndromes due to insufficient evidence. Teasel *et*. *al*. (2003) report that the evidence linking acupuncture to post-stroke ADL recovery is conflicting [97]. Our current review corresponds with previous uncertainties. There was limited evidence to show that acupuncture can benefit older stroke survivors and further research is required.

### Caregiver training

Stroke guidelines acknowledge the insufficient evidence behind the benefits of caregiver training as part of stroke rehabilitation, but do promote carer involvement in patient rehabilitation as good practice [4–5]. Our current review identified only one study exploring the impact of caregiver training upon older stroke survivors ADL. This study was sufficiently large, and demonstrated benefits in ADL recovery, but these were short term [31]. Therefore, caregiver training may be beneficial, but further research is required to examine this intervention further.

### CIMT

Our current review found little evidence to support the use of CIMT with older stroke survivors, similar to the findings of Veerbeek *et*. *al*. (2014) [98]. Several stroke rehabilitation guidelines recommend CIMT to improve function of impaired upper limbs [3–5, 99]. In view of the evidence, SIGN (2010) specifically state that *“Constraint induced movement therapy may be considered for carefully selected individuals with at least 10 degrees of finger extension*, *intact balance and cognition”* (p20) [5]. RCP (2016) also explain that the benefits of CIMT often relate to arm function only and within the confines of the activities used within the intervention [4]. Similar to other stroke rehabilitation interventions, CIMT appears most effective when effectiveness is measured in terms of its immediate effect on physiological variables, such as muscle strength. But such benefits do not appear to be associated with improvements in more comprehensive or global outcomes of ADL or disability.

### Device assisted physiotherapy

Our current review identified limited evidence to support the use of device assisted physiotherapy to enhance older stroke survivors stroke rehabilitation. Our findings are in line with other reviews and guidelines [4, 98]. The use of robotic devices has been recommended by Teasel *et*. *al*. (2003) as they considered this approach beneficial for those with impaired arm function [97], but this recommendation was based on achieving improved arm function, not improved ADL. Conversely, as a result of the overall low quality of evidence behind robot assisted movement therapies the RCP (2016) guidelines stipulate that this type of therapy should only be offered as an adjunct to conventional therapy and within the context of a clinical trial [4].

### Music therapy

Only one study explored music therapy in relation to older stroke survivors ADL [42] and no evidence was found to support use of this intervention. Music therapy has been explored previously within neuro-rehabilitation and reviews have identified several benefits such as improved motor function, language and mood [100–102]. Nevertheless, their efficacy with older stroke survivors, and/or impact on global ADL or disability, remains unknown.

### Nerve stimulation

Current stroke guidelines have all noted uncertainties surrounding the efficacy of nerve stimulation [4–5, 97, 99]. Evidence has shown that while nerve stimulation techniques can improve specific impairments, such as muscle strength or gait, these improvements do not lead to significant improvements in ADL or disability [4]. Within this review, limited evidence was identified supporting the efficacy of nerve stimulation upon older stroke survivors ADL. Therefore nerve stimulation may benefit older stroke survivors ADL, but the quality of evidence is weak. The number of included studies focusing exclusively upon older stroke survivors is small, making it difficult to sub-divide studies into those focusing on specific types of stimulation or use of stimulation in different locations (e.g. upper or lower body). Reviews which have included adult participants of all ages suggest the best evidence behind nerve stimulation may be found in its use for upper limb impairments [98, 103]. However, due to inconsistencies, current evidence remains insufficient to make any recommendations [5].

### Occupational therapy

There was some evidence to show that additional OT can benefit older stroke survivors ADL. This is consistent with the reporting of uncertainties regarding the effects of increased intensity or frequency of OT [4–5]. This review found no evidence to suggest that one OT approach is more beneficial than anther, consistent with the review by Teasel *et*. *al*. (2003) [97]. All guidelines recommend ADL focused OT as an important feature of stroke rehabilitation, but acknowledge that optimal intensity and/or duration is yet to be determined [4–5].

### Optical

Within the Royal College of Physicians (2016) stroke guidelines, optical interventions such as prism glasses are recommended for stroke survivors with visual neglect [4]. This said, it is noted that the evidence is very limited and that patient participation in such interventions would be most beneficial within the context of a clinical trial [4], a finding echoed by Langhorne, Bernhardt & Kwakkel (2011) [99]. A Cochrane review of interventions targeted at spatial neglect following stroke concluded that there was insufficient evidence to support the use of these interventions to improve disability and ADL [104]. However, the evidence was more promising for specific visual neglect measures [104]. This current review identified no evidence to support the use of these interventions. However, a combination of few studies and small sample sizes may obscure any potential positive impact from these interventions. As suggested by Bowen, Lincoln & Dewey (2007) [104], we also recommend further research involving larger high quality RCTs.

### Physiotherapy

It has been reported that many PT interventions such as balance exercises, gait training, and fitness training do lead to benefits in their respective objectives i.e. improved balance, gait, cardiovascular fitness, but rarely lead to improvement in more global measures such as ADL and disability [97]. Veerbeek *et*. *al*. (2014) systematically reviewed 467 RCTS involving PT interventions to examine their efficacy in stroke rehabilitation [98]. The strongest evidence supported task specific functions and activities which are repeated at high intensity [98]. However, few global outcomes were reported; outcomes included muscle, joint, bone and sensory function, gait pattern, balance and walking [98]. Benefits in terms of basic ADL were reported to arise from interventions involving activity-based balance training, assisted gait training and VR training for paretic arm [98].

This current review identified limited evidence to demonstrate that additional PT can benefit older stroke survivors’ ADL in comparison to usual care. When restricted to older stroke survivors, and those reporting global measure of ADL or disability, the resulting number of included studies is considerably smaller than those cited in reviews such as that by Veerbeek *et*. *al*. (2014) [98]. Nevertheless, we found some evidence suggesting older stroke survivors may benefit from increased PT.

### Psychological therapies

Within the guidelines for stroke rehabilitation it is recommended that all stroke survivors be considered and offered psychological care, and not just offer to those with an identified mental health disorder [4]. This recommendation is based upon good practice, possibly due to the frequent development of post-stroke depression [4–5]. In this review limited evidence was identified to show that psychological therapies benefit older stroke survivors. Therefore, psychological therapies may benefit older stroke survivors ADL but the evidence for this is weak and requires further investigation.

### Self-management education

Self-management is reported to be capable of influencing function and social participation, and recommended for stroke survivors [4]. This review identified limited evidence to show that such interventions can benefit older stroke survivors. The quality of included studies varied from low to very low and therefore the evidence for this is weak. Further research focusing exclusively upon older stroke survivors and global outcomes is recommended.

### Videogames

Current guidelines report that the evidence behind virtual reality as a stroke rehabilitation approach is weak to moderate [4–5]. A Cochrane review by Laver *et*. *al*. (2015) suggests that virtual reality can benefit upper limb impairments and ADL, but that evidence is limited to younger stroke survivors and those who are more than one-year post-stroke [105]. In addition to larger high quality RCTs, it has been recommended that research focus upon identifying what the important elements of virtual reality are, and if benefits can be sustained in the long term [105]. Another review suggests that the best evidence lies behind the efficacy of virtual reality upon gait improvement [97]. Based on the evidence generated in this current review it is not possible to recommend virtual reality for older stroke survivors’ rehabilitation. However, based on evidence identified by studies involving slightly younger stroke survivors, further research involving appropriately sized high quality RCTs is warranted.

### Wheelchair use

Only one study was identified which investigated if self-propulsion of a wheelchair improved ADL recovery against non-self-propulsion, and there was no evidence of the efficacy of this approach. Current recommendations do not include such an intervention but do recommend the use of wheelchairs in those with impaired mobility to promote independence [4–5].

### Limitations

This review has several limitations which must be considered alongside our findings. Firstly, we did not involve patients or carers in the Delphi process, and our identified critical outcomes may not reflect patient and carer preferences. Due to study heterogeneity and insufficient data this review has been limited to narrative analysis only. While describing comparisons between studies is important, it has potential for researcher bias through the imposition of the researchers own subjective ideas about the findings and lacks the rigour of qualitative and objective analysis. Although we used the GRADE criteria recommended by the Cochrane Collaboration this also introduced a degree of subjectivity. This means our results should be interpreted cautiously. We also cannot exclude the possibility that this review has omitted important studies.We have not searched the grey literature and our search strategy focused exclusively on identifying systematic reviews which may have resulted in omission of some trials, particularly those more recently published. However, our comprehensive strategy and the checking of reference lists and published clinical guidelines does go some way in reducing this risk. Categories of non-pharmacological interventions were developed in a somewhat arbitrary fashion. It could be argued that interventions exploring nerve stimulation devices, often delivered by trained physiotherapists, could be considered an alternative physiotherapy approach, as opposed to a category in its own right. Our decisions regarding categorising interventions were largely pragmatic and aimed to organise and present findings in a meaningful way. However, the findings should be interpreted with caution since the interventions lack specificity. We also do not consider the preferences of patients and their carers regarding intervention types. Little work has been done in this area and acceptability of these non-pharmacological approaches are unknown.

Finally, our review is restricted by the significant lack of published studies which met our age criteria (mean age≥ 65 years) and presented results using a global measure of ADL and/or disability. Age-based criteria allow us to examine the evidence as it specifically relates to older adults, but it risks excluding interventions which may be beneficial but have not been adequately tested in an older population. The impact of age as a modifier of treatment effect for many of the interventions examined is unknown. Similarly, the exclusion of so many studies due to lack of global outcome measures again risks excluding worthy interventions which may have demonstrated efficacy had a global outcome been assessed. Additionally, this review uncovered a number of methodological and reporting problems, making the ascertainment of the evidence challenging. Small sample sizes and failure to adequately report details regarding participant selection, randomisation, allocation concealment and data analysis, especially the management of missing data, led to many studies being deemed high risk of bias. One important challenge regarding RCTs involving non-pharmacological treatments is the lack of participant blinding. Although blinding of non-pharmacological treatments is challenging, reviews do highlight many creative approaches to doing so [106]. However, opinions regarding the importance of this are divided. Lack of patient blinding in RCTs presents opportunity for bias, particularly for subjective outcomes [106] such as those explored in the present manuscript. However, concerns have been raised about false negative results arising from RCTs involving non-pharmacological treatments as a result of blinded participants [107]. It is argued that what factors blinding controls for may be an integral component of non-pharmacological therapy [107]. For example, the additional care an intervention participant may receive as part of their acupuncture treatment may contribute towards overall benefit of the treatment [107]. In pharmacological RCTs this additional care would be considered incidental and would be controlled for through provision of similar care to control participants [106–107]. However, it has been argued that this takes away from some of the benefits non-pharmacological treatments bring, and therefore leads to findings of no-benefit [107]. It may be prudent for future work to explore the role of incidental and placebo effects in non-pharmacological treatments for stroke survivors to enhance our confidence in future results.

## Conclusion

Due to the substantial heterogeneity, moderate to high risk of biases, and insufficient data provided, this review has had to make recommendations based on narrative analysis only.

Limited evidence suggests additional physiotherapy or occupational therapy may benefit older stroke survivors ADL. Very limited evidence also suggests acupuncture, self-management education, psychological therapies, nerve stimulation, CIMT, and caregiver training may benefit older stroke survivors ADL.

However, the current evidence base is limited by the low number and quality of studies. This review revealed a distinct lack of evidence behind the use of non-pharmacological interventions for stroke survivors aged 65 years and older. Of studies which did involve those aged 65 and older, evidence is limited by poor study designs and inadequate study reporting. Therefore, we also recommend that future studies explore these interventions exclusively in older adult populations, and ensure studies are adequately reported both in terms of methodological detail but also in terms of their outcomes.

## Supporting information

S1 Checklist PRISMA (DOC)

S1 Table Database search strategies. (DOCX)

S2 Table References of studies included in the systematic review. (DOCX)

S3 Table Description of all included interventions. (DOCX)

## Abbreviations

ADL: Activities of Daily Living
BI: Barthel Index
CIMT: Constraint Induced Movement Therapy
FAI: Frenchay Activities Index
FIM: Functional Independence Measure
GRADE: Grading of Recommendations, Development and Evaluation
IADL: Instrumental Activities of Daily Living
ONTOP: Optimal Evidence-Based Non-Drug Therapies in Older Persons
OT: Occupational Therapy
PT: Physiotherapy
SENATOR: Software Engine for the Assessment and Optimisation of Drug and Non-Drug Therapies in Older Persons
TENS: Trans-electrical Nerve stimulation
