# Supplementary material for: Non-pharmacological interventions for the improvement of post-stroke activities of daily living and disability amongst older stroke survivors: A systematic review
Source: PLoS One. 2018 Oct 4;13(10):e0204774. doi: 10.1371/journal.pone.0204774 (PMC6171865; doi:10.1371/journal.pone.0204774)
Supplement: S1 Table — (DOCX) [file pone.0204774.s002.docx]

# S1 Database Search Strategies

| **Database** | **Search Strategy** |
| --- | --- |
| Pubmed | (((((("Cerebrovascular Disorders"[Mesh]) OR "Basal Ganglia Cerebrovascular Disease"[Mesh]) OR "Brain Ischemia"[Mesh]) OR "Intracranial Embolism and Thrombosis"[Mesh]) OR "Intracranial Hemorrhages"[Mesh]) OR "Stroke, Lacunar"[Mesh] OR "Stroke"[Mesh] OR stroke OR poststroke OR post-stroke OR cerebrovasc* OR (cerebral vasc*)) AND ((MEDLINE[Title/Abstract] OR (systematic[Title/Abstract] AND review[Title/Abstract] OR metaanalysis[Publication Type])))  Dates Searched: 1809 – April 2018 |
| Cinahl | (MH "Cerebrovascular Disorders+") OR (MH "Basal Ganglia Cerebrovascular Disease+") OR (MH "Cerebral Ischemia+") OR ( (MH "Intracranial Embolism and Thrombosis+") ) OR (MH "Intracranial Hemorrhage+") OR (MH "Stroke, Lacunar") OR (MH "Stroke+") OR ( TI ( stroke or poststroke or post-stroke or cerebrovasc* cerebral vasc* ) OR AB ( stroke or poststroke or post-stroke or cerebrovasc* cerebral vasc* ) ) Limiters: Human; Publication Type: Meta Analysis, Systematic Review  Dates Searched: 1981 – April 2018 |
| Cochrane | 1) MeSH descriptor: [Cerebrovascular Disorders] explode all trees 2) MeSH descriptor: [Basal Ganglia Cerebrovascular Disease] explode all trees 3) MeSH descriptor: [Brain Ischemia] explode all trees 4) MeSH descriptor: [Intracranial Embolism and Thrombosis] explode all trees 5) MeSH descriptor: [Intracranial Hemorrhages] explode all trees 6) MeSH descriptor: [Stroke, Lacunar] explode all trees 7) MeSH descriptor: [Stroke] explode all trees 8) stroke or poststroke or post-stroke or cerebrovasc* or cerebral vasc*:ti,ab,kw (Word variations have been searched) 9) #1 or #2 or #3 or #4 or #5 or #6 or #7 or #8 10) #9 in Cochrane Reviews (Reviews only)  Dates Searched: 1993 – April 2018 |
| Embase | 1) cerebrovascular disease/  2) exp brain embolism/  3) exp occlusive cerebrovascular disease/  4) cerebral ischemia/  5) brain hemorrhage/  6) exp cerebrovascular accident/  7) (stroke or poststroke or post-stroke or cerebrovasc* or cerebral vasc*).tw.  8) 1 or 2 or 3 or 4 or 5 or 6 or 7  9) limit 8 to (human and (meta analysis or "systematic review"))  Dates Searched: 1974 – April 2018 |
| PsycInfo | 1) Exp Cerebrovascular Disorders/  2) exp Cerebrovascular Accidents/ or exp Cerebral Ischemia/  3) exp Cerebral Hemorrhage/  4) (stroke or poststroke or post-stroke or cerebrovasc* or cerebral vasc*).tw.  5) 1 or 2 or 3 or 4  6) limit 5 to (human and ("0830 systematic review" or 1200 meta analysis))  Dates Searched: 1806 – April 2018 |
