# Supplementary material for: Non-pharmacological interventions for the improvement of post-stroke activities of daily living and disability amongst older stroke survivors: A systematic review
Source: PLoS One. 2018 Oct 4;13(10):e0204774. doi: 10.1371/journal.pone.0204774 (PMC6171865; doi:10.1371/journal.pone.0204774)
Supplement: S2 Table — (DOCX) [file pone.0204774.s003.docx]

# S2 References of Studies Included in the Systematic Review

| Askim, T., Morkved, S., Engen, A., Roos, K., Aas, T., & Indredavik, B. (2010). Effects of a community-based intensive motor training program combined with early supported discharge after treatment in a comprehensive stroke unit: A randomized, controlled trial. *Stroke, 41*(8), 1697-1703. doi:10.1161/STROKEAHA.110.584284 [doi] |
| --- |
| Bagley, P., Hudson, M., Forster, A., Smith, J., & Young, J. (2005). A randomized trial evaluation of the oswestry standing frame for patients after stroke. *Clinical Rehabilitation, 19*(4), 354-364. |
| Barrett, J., Watkins, C., Plant, R., Dickinson, H., Clayton, L., Sharma, A., . . . Flynn, A. (2001). The COSTAR wheelchair study: A two-centre pilot study of self-propulsion in a wheelchair in early stroke rehabilitation. *Clinical Rehabilitation, 15*(1), 32-41. |
| Bradley, L., Hart, B. B., Mandana, S., Flowers, K., Riches, M., & Sanderson, P. (1998). Electromyographic biofeedback for gait training after stroke. *Clinical Rehabilitation, 12*(1), 11-22. |
| Braun, S. M., Beurskens, A. J., Kleynen, M., Oudelaar, B., Schols, J. M., & Wade, D. T. (2012). A multicenter randomized controlled trial to compare subacute ‘treatment as usual’with and without mental practice among persons with stroke in dutch nursing homes. *Journal of the American Medical Directors Association, 13*(1), 85. e1-85. e7. |
| Chiu, C. W., & Man, D. W. (2004). The effect of training older adults with stroke to use home-based assistive devices. *OTJR: Occupation, Participation and Health, 24*(3), 113-120. |
| Clark, M. S., Rubenach, S., & Winsor, A. (2003). A randomized controlled trial of an education and counselling intervention for families after stroke. *Clinical Rehabilitation, 17*(7), 703-712. |
| Corr, S., & Bayer, A. (1995). Occupational therapy for stroke patients after hospital discharge—a randomized controlled trial. *Clinical Rehabilitation, 9*(4), 291-296. |
| de Sèze, M., Wiart, L., Bon-Saint-Côme, A., Debelleix, X., de Sèze, M., Joseph, P., . . . Barat, M. (2001). Rehabilitation of postural disturbances of hemiplegic patients by using trunk control retraining during exploratory exercises. *Archives of Physical Medicine and Rehabilitation, 82*(6), 793-800. |
| Dickstein, R., Hocherman, S., Pillar, T., & Shaham, R. (1986). Stroke rehabilitation: Three exercise therapy approaches. *Physical Therapy, 66*(8), 1233-1238. |
| Donkervoort, M., Dekker, J., Stehmann-Saris, F. C., & Deelman, B. G. (2001). Efficacy of strategy training in left hemisphere stroke patients with apraxia: A randomised clinical trial. *Neuropsychological Rehabilitation, 11*(5), 549-566. |
| Duncan, P., Richards, L., Wallace, D., Stoker-Yates, J., Pohl, P., Luchies, C., . . . Studenski, S. (1998). A randomized, controlled pilot study of a home-based exercise program for individuals with mild and moderate stroke. *Stroke, 29*(10), 2055-2060. |
| Duncan, P., Studenski, S., Richards, L., Gollub, S., Lai, S. M., Reker, D., . . . Johnson, D. (2003). Randomized clinical trial of therapeutic exercise in subacute stroke. *Stroke, 34*(9), 2173-2180. doi:10.1161/01.STR.0000083699.95351.F2 [doi] |
| Ertel, K., Glymour, M., Glass, T., & Berkman, L. (2007). Frailty modifies effectiveness of psychosocial intervention in recovery from stroke. *Clinical Rehabilitation, 21*(6), 511-522. |
| Forster, A., & Young, J. (1996). Specialist nurse support for patients with stroke in the community: A randomised controlled trial. *BMJ (Clinical Research Ed.), 312*(7047), 1642-1646. |
| Franceschini, M., Carda, S., Agosti, M., Antenucci, R., Malgrati, D., Cisari, C., & Gruppo Italiano Studio Allevio Carico Ictus. (2009). Walking after stroke: What does treadmill training with body weight support add to overground gait training in patients early after stroke?: A single-blind, randomized, controlled trial. *Stroke, 40*(9), 3079-3085. doi:10.1161/STROKEAHA.109.555540 [doi] |
| Galvin, R., Cusack, T., O'Grady, E., Murphy, T. B., & Stokes, E. (2011). Family-mediated exercise intervention (FAME): Evaluation of a novel form of exercise delivery after stroke. *Stroke, 42*(3), 681-686. doi:10.1161/STROKEAHA.110.594689 [doi] |
| Gelber, D. A., Josefczyk, B., Herrman, D., Good, D. C., & Verhulst, S. J. (1995). Comparison of two therapy approaches in the rehabilitation of the pure motor hemiparetic stroke patient. *Journal of Neurologic Rehabilitation, 9*(4), 191-196. |
| Gilbertson, L., Langhorne, P., Walker, A., Allen, A., & Murray, G. D. (2000). Domiciliary occupational therapy for patients with stroke discharged from hospital: Randomised controlled trial. *BMJ (Clinical Research Ed.), 320*(7235), 603-606. |
| Glasgow Augmented Physiotherapy Study Group. (2004). Can augmented physiotherapy input enhance recovery of mobility after stroke? A randomized controlled trial. *Clinical Rehabilitation, 18*(5), 529. |
| Glass, T. A., Berkman, L. F., Hiltunen, E. F., Furie, K., Glymour, M. M., Fay, M. E., & Ware, J. (2004). The families in recovery from stroke trial (FIRST): Primary study results. *Psychosomatic Medicine, 66*(6), 889-897. |
| Gosman-Hedstrom, G., Claesson, L., Klingenstierna, U., Carlsson, J., Olausson, B., Frizell, M., . . . Blomstrand, C. (1998). Effects of acupuncture treatment on daily life activities and quality of life: A controlled, prospective, and randomized study of acute stroke patients. *Stroke, 29*(10), 2100-2108. |
| Green, J., Forster, A., Bogle, S., & Young, J. (2002). Physiotherapy for patients with mobility problems more than 1 year after stroke: A randomised controlled trial. *The Lancet, 359*(9302), 199-203. |
| Guidetti, S., Andersson, K., Andersson, M., Tham, K., & Koch, L. V. (2010). Client-centred self-care intervention after stroke: A feasibility study. *Scandinavian Journal of Occupational Therapy, 17*(4), 276-285. |
| Guidetti, S., & Ytterberg, C. (2011). A randomised controlled trial of a client-centred self-care intervention after stroke: A longitudinal pilot study. *Disability and Rehabilitation, 33*(6), 494-503. |
| Hopwood, V., Lewith, G., Prescott, P., & Campbell, M. (2008). Evaluating the efficacy of acupuncture in defined aspects of stroke recovery. *Journal of Neurology, 255*(6), 858-866. |
| Hsieh, R., Wang, L., & Lee, W. (2007). Additional therapeutic effects of electroacupuncture in conjunction with conventional rehabilitation for patients with first-ever ischaemic stroke. *Journal of Rehabilitation Medicine, 39*(3), 205-211. |
| Johansson, B. B., Haker, E., von Arbin, M., Britton, M., Langstrom, G., Terent, A., . . . Swedish Collaboration on Sensory Stimulation After Stroke. (2001). Acupuncture and transcutaneous nerve stimulation in stroke rehabilitation: A randomized, controlled trial. *Stroke, 32*(3), 707-713. |
| Johansson, K., Lindgren, I., Widner, H., Wiklund, I., & Johansson, B. B. (1993). Can sensory stimulation improve the functional outcome in stroke patients? *Neurology, 43*(11), 2189-2192. |
| Johnston, M., Bonetti, D., Joice, S., Pollard, B., Morrison, V., Francis, J. J., & MacWalter, R. (2007). Recovery from disability after stroke as a target for a behavioural intervention: Results of a randomized controlled trial. *Disability and Rehabilitation, 29*(14), 1117-1127. |
| Jongbloed, L., Stacey, S., & Brighton, C. (1989). Stroke rehabilitation: Sensorimotor integrative treatment versus functional treatment. *American Journal of Occupational Therapy, 43*(6), 391-397. |
| Kalra, L., Evans, A., Perez, I., Melbourn, A., Patel, A., Knapp, M., & Donaldson, N. (2004). Training carers of stroke patients: Randomised controlled trial. *BMJ (Clinical Research Ed.), 328*(7448), 1099. doi:10.1136/bmj.328.7448.1099 [doi] |
| Kalra, L., Perez, I., Gupta, S., & Wittink, M. (1997). The influence of visual neglect on stroke rehabilitation. *Stroke, 28*(7), 1386-1391. |
| Kim, S. M., Han, E. Y., Kim, B. R., & Hyun, C. W. (2016). Clinical application of circuit training for subacute stroke patients: a preliminary study. *Journal of physical therapy science*, *28*(1), 169 |
| Kwakkel, G., Wagenaar, R. C., Twisk, J. W., Lankhorst, G. J., & Koetsier, J. C. (1999). Intensity of leg and arm training after primary middle-cerebral-artery stroke: A randomised trial. *The Lancet, 354*(9174), 191-196. |
| Kwakkel, G., Kollen, B. J., & Wagenaar, R. C. (2002). Long term effects of intensity of upper and lower limb training after stroke: A randomised trial. *Journal of Neurology, Neurosurgery, and Psychiatry, 72*(4), 473-479. |
| Landi, F., Cesari, M., Onder, G., Tafani, A., Zamboni, V., & Cocchi, A. (2006). Effects of an occupational therapy program on functional outcomes in older stroke patients. *Gerontology, 52*(2), 85-91. doi:90953 [pii] |
| Langhammer, B., & Stanghelle, J. K. (2000). Bobath or motor relearning programme? A comparison of two different approaches of physiotherapy in stroke rehabilitation: A randomized controlled study. *Clinical Rehabilitation, 14*(4), 361-369. |
| Langhammer, B., & Stanghelle, J. K. (2003). Bobath or motor relearning programme? A follow-up one and four years post stroke. *Clinical Rehabilitation, 17*(7), 731-734. |
| Lee, G. (2013). Effects of training using video games on the muscle strength, muscle tone, and activities of daily living of chronic stroke patients. *Journal of Physical Therapy Science, 25*(5), 595-597. |
| Lincoln, N. B., & Flannaghan, T. (2003). Cognitive behavioral psychotherapy for depression following stroke: A randomized controlled trial. *Stroke, 34*(1), 111-115. |
| Lincoln, N. B., Parry, R. H., & Vass, C. D. (1999). Randomized, controlled trial to evaluate increased intensity of physiotherapy treatment of arm function after stroke. *Stroke, 30*(3), 573-579. |
| Liu, K. P., Balderi, K., Leung, T. L. F., Yue, A. S. Y., Lam, N. C. W., Cheung, J. T. Y., ... & Mok, V. C. T. (2016a). A randomized controlled trial of self‐regulated modified constraint‐induced movement therapy in sub‐acute stroke patients. *European journal of neurology*, *23*(8), 1351-1360. |
| Liu, C. H., Hsieh, Y. T., Tseng, H. P., Lin, H. C., Lin, C. L., Wu, T. Y., ... & Zhang, H. (2016b). Acupuncture for a first episode of acute ischaemic stroke: an observer-blinded randomised controlled pilot study. *Acupuncture in Medicine*, *34*(5), 349-355. |
| Logan, P., Ahern, J., Gladman, J., & Lincoln, N. (1997). A randomized controlled trial of enhanced social service occupational therapy for stroke patients. *Clinical Rehabilitation, 11*(2), 107-113. |
| Logan, P. A., Gladman, J. R., Avery, A., Walker, M. F., Dyas, J., & Groom, L. (2004). Randomised controlled trial of an occupational therapy intervention to increase outdoor mobility after stroke. *BMJ (Clinical Research Ed.), 329*(7479), 1372-1375. doi:bmj.38264.679560.8F [pii] |
| Macdonell, R. A., Triggs, W., Leikauskas, J., Bourque, M., Robb, K., Day, B., & Shahani, B. (1994). Functional electrical stimulation to the affected lower limb and recovery after cerebral infarction. *Journal of Stroke and Cerebrovascular Diseases, 4*(3), 155-160. |
| Masiero, S., Celia, A., Rosati, G., & Armani, M. (2007). Robotic-assisted rehabilitation of the upper limb after acute stroke. *Archives of Physical Medicine and Rehabilitation, 88*(2), 142-149. |
| Min, M., Xin, C., Yuefeng, C., Ping, R., & Jian, L. (2008). Stage-oriented comprehensive acupuncture treatment plus rehabilitation training for apoplectic hemiplegia. *Journal of Traditional Chinese Medicine, 28*(2), 90-93. |
| Mizuno, K., Tsuji, T., Takebayashi, T., Fujiwara, T., Hase, K., & Liu, M. (2011). Prism adaptation therapy enhances rehabilitation of stroke patients with unilateral spatial neglect: A randomized, controlled trial. *Neurorehabilitation and Neural Repair, 25*(8), 711-720. |
| Morris, J. H., van Wijck, F., Joice, S., Ogston, S. A., Cole, I., & MacWalter, R. S. (2008). A comparison of bilateral and unilateral upper-limb task training in early poststroke rehabilitation: A randomized controlled trial. *Archives of Physical Medicine and Rehabilitation, 89*(7), 1237-1245. |
| Ng, M. F., Tong, R. K., & Li, L. S. (2008). A pilot study of randomized clinical controlled trial of gait training in subacute stroke patients with partial body-weight support electromechanical gait trainer and functional electrical stimulation: Six-month follow-up. *Stroke, 39*(1), 154-160. doi:STROKEAHA.107.495705 [pii] |
| Nir, Z., Zolotogorsky, Z., & Sugarman, H. (2004). Structured nursing intervention versus routine rehabilitation after stroke. *American Journal of Physical Medicine & Rehabilitation, 83*(7), 522-529. doi:00002060-200407000-00005 [pii] |
| Park, J., White, A. R., James, M. A., Hemsley, A. G., Johnson, P., Chambers, J., & Ernst, E. (2005). Acupuncture for subacute stroke rehabilitation: A sham-controlled, subject-and assessor-blind, randomized trial. *Archives of Internal Medicine, 165*(17), 2026-2031. |
| Parker, C., Gladman, J. R., Drummond, A. E., Dewey, M., Lincoln, N., Barer, D., . . . Radford, K. (2001). A multicentre randomized controlled trial of leisure therapy and conventional occupational therapy after stroke. *Clinical Rehabilitation, 15*(1), 42-52. |
| Pei, J., Sun, L., Chen, R., Zhu, T., Qian, Y., & Yuan, D. (2001). The effect of electro-acupuncture on motor function recovery in patients with acute cerebral infarction: A randomly controlled trial. *Journal of Traditional Chinese Medicine = Chung i Tsa Chih Ying Wen Pan, 21*(4), 270-272. |
| Rabadi, M., Galgano, M., Lynch, D., Akerman, M., Lesser, M., & Volpe, B. (2008). A pilot study of activity-based therapy in the arm motor recovery post stroke: A randomized controlled trial. *Clinical Rehabilitation, 22*(12), 1071-1082. |
| Raglio, A., Zaliani, A., Baiardi, P., Bossi, D., Sguazzin, C., Capodaglio, E., ... & Imbriani, M. (2017). Active music therapy approach for stroke patients in the post-acute rehabilitation. *Neurological Sciences*, *38*(5), 893-897. |
| Rodgers, H., Atkinson, C., Bond, S., Suddes, M., Dobson, R., & Curless, R. (1999). Randomized controlled trial of a comprehensive stroke education program for patients and caregivers. *Stroke, 30*(12), 2585-2591. |
| Rydwik, E., Eliasson, S., & Akner, G. (2006). The effect of exercise of the affected foot in stroke patients-a randomized controlled pilot trial. *Clinical Rehabilitation, 20*(8), 645-655. |
| Sackley, C., Wade, D. T., Mant, D., Atkinson, J. C., Yudkin, P., Cardoso, K., . . . Reel, K. (2006). Cluster randomized pilot controlled trial of an occupational therapy intervention for residents with stroke in UK care homes. *Stroke, 37*(9), 2336-2341. doi:01.STR.0000237124.20596.92 [pii] |
| Schuler, M. S., Durdak, C., Hösl, N. M., Klink, A., Hauer, K. A., Oster, P., & Du, X. (2005). Acupuncture treatment of geriatric patients with ischemic stroke: A randomized, double‐controlled, single‐blind study. *Journal of the American Geriatrics Society, 53*(3), 549-550. |
| Sivenius, J., Pyorala, K., Heinonen, O. P., Salonen, J. T., & Riekkinen, P. (1985). The significance of intensity of rehabilitation of stroke--a controlled trial. *Stroke, 16*(6), 928-931. |
| Smith, J., Forster, A., & Young, J. (2004). A randomized trial to evaluate an education programme for patients and carers after stroke. *Clinical Rehabilitation, 18*(7), 726-736. |
| Sonde, L., Gip, C., Ferneaus, S., Nilsson, C., & Viitanen, M. (1998). Stimulation with low frequency (1.7 hz) transcutaneous electric nerve stimulation (low-tens) increases motor function of the post-stroke paretic arm. *Scandinavian Journal of Rehabilitation Medicine, 30*(2), 95-100. |
| Sonde, L., Kalimo, H., Fernaeus, S., & Viitanen, M. (2000). Low TENS treatment on post-stroke paretic arm: A three-year follow-up. *Clinical Rehabilitation, 14*(1), 14-19. |
| Studenski, S., Duncan, P. W., Perera, S., Reker, D., Lai, S. M., & Richards, L. (2005). Daily functioning and quality of life in a randomized controlled trial of therapeutic exercise for subacute stroke survivors. *Stroke, 36*(8), 1764-1770. doi:01.STR.0000174192.87887.70 [pii] |
| Sunderland, A., Tinson, D. J., Bradley, E. L., Fletcher, D., Langton Hewer, R., & Wade, D. T. (1992). Enhanced physical therapy improves recovery of arm function after stroke. A randomised controlled trial. *Journal of Neurology, Neurosurgery, and Psychiatry, 55*(7), 530-535. |
| Sze, F. K., Wong, E., Yi, X., & Woo, J. (2002). Does acupuncture have additional value to standard poststroke motor rehabilitation? *Stroke, 33*(1), 186-194. |
| Tsang, M., Sze, K., & Fong, K. (2009). Occupational therapy treatment with right half-field eye-patching for patients with subacute stroke and unilateral neglect: A randomised controlled trial. *Disability and Rehabilitation, 31*(8), 630-637. |
| van Vliet, P. M., Lincoln, N. B., & Foxall, A. (2005). Comparison of bobath based and movement science based treatment for stroke: A randomised controlled trial. *Journal of Neurology, Neurosurgery, and Psychiatry, 76*(4), 503-508. doi:76/4/503 [pii] |
| Wade, D. T., Collen, F. M., Robb, G. F., & Warlow, C. P. (1992). Physiotherapy intervention late after stroke and mobility. *BMJ (Clinical Research Ed.), 304*(6827), 609-613. |
| Walker, M., Drummond, A., & Lincoln, N. (1996). Evaluation of dressing practice for stroke patients after discharge from hospital: A crossover design study. *Clinical Rehabilitation, 10*(1), 23-31. |
| Walker, M., Gladman, J., Lincoln, N., Siemonsma, P., & Whiteley, T. (1999). Occupational therapy for stroke patients not admitted to hospital: A randomised controlled trial. *The Lancet, 354*(9175), 278-280. |
| Watkins, C. L., Auton, M. F., Deans, C. F., Dickinson, H. A., Jack, C. I., Lightbody, C. E., . . . Leathley, M. J. (2007). Motivational interviewing early after acute stroke: A randomized, controlled trial. *Stroke, 38*(3), 1004-1009. doi:01.STR.0000258114.28006.d7 [pii] |
| Wiart, L., Saint Côme, A. B., Debelleix, X., Petit, H., Joseph, P. A., Mazaux, J. M., & Barat, M. (1997). Unilateral neglect syndrome rehabilitation by trunk rotation and scanning training. *Archives of Physical Medicine and Rehabilitation, 78*(4), 424-429. |
| Wu, C., Chen, C., Tsai, W., Lin, K., & Chou, S. (2007). A randomized controlled trial of modified constraint-induced movement therapy for elderly stroke survivors: Changes in motor impairment, daily functioning, and quality of life. *Archives of Physical Medicine and Rehabilitation, 88*(3), 273-278. |
| Zhu, Y., Zhang, L., Ouyang, G., Meng, D., Qian, K., Ma, J., & Wang, T. (2013). Acupuncture in subacute stroke: No benefits detected. *Physical Therapy, 93*(11), 1447-1455. |
